# Within‐individual leaf trait response to local light availability and biodiversity in a subtropical forest experiment

**DOI:** 10.1002/ecy.70160

**Published:** 2025-07-18

**Authors:** Tobias Proß, Helge Bruelheide, Sylvia Haider

**Affiliations:** ^1^ Institute of Biology, Geobotany and Botanical Garden Martin Luther University Halle‐Wittenberg Halle Saxony‐Anhalt Germany; ^2^ German Centre for Integrative Biodiversity Research (iDiv) Leipzig Saxony Germany; ^3^ Leuphana University of Lüneburg Institute of Ecology Lüneburg Lower Saxony Germany

**Keywords:** BEF‐China, FieldSpec, leaf habit, leaf trait variation, leaf traits, light availability, NIRS, phenotypic plasticity, subtropical forest, Vis/NIR spectroscopy

## Abstract

Leaf traits are important indicators of ecosystem functions. Trait values can vary widely between species, and a considerable amount of variation also occurs within species. However, within‐individual variation is often neglected due to the limitations of traditional measurement tools. Many leaf trait values respond to light availability, which, in turn, is affected by the surrounding vegetation. Additionally, there is a strong within‐individual light gradient, especially in tree canopies. In the BEF‐China (Biodiversity–Ecosystem Functioning China) subtropical forest plantation, we analyzed how leaf trait values respond to light availability and neighboring tree species richness at the within‐individual level. We sampled across the vertical light gradient formed by neighboring trees planted at varying diversity levels from monocultures to 24‐species mixtures. We closely paired the leaf samples with sensor‐based measurements of light availability. We used visible and near‐infrared spectroscopy (spectral range: 350–2500 nm) to predict 14 leaf traits across 4981 leaves from 15 native tree species. Using a key feature of spectroscopy—deriving multiple leaf traits from a single spectral measurement of a sample—we assessed all traits simultaneously at the leaf level. We investigated whether an individual tree's direct neighbor or the surrounding tree species richness had a stronger influence on the light–trait relationship. Most trait values responded to light availability, though this response differed between deciduous and evergreen species. We found that tree species richness and a tree's direct neighbor could modify the light–trait relationship at the individual level. In some instances, a focal tree's direct neighbor influenced its leaf trait values more than the tree species richness in its local neighborhood. Specifically, in conspecific tree pairs of evergreens, specific leaf area and leaf nitrogen displayed a stronger response to changing light conditions. This response to light availability suggests a mechanism for avoiding within‐species competition that is observable at the within‐individual level. Our results show that biodiversity influences ecosystem functions through its effects on within‐individual leaf trait variation. The fact that the interplay between light availability, biodiversity, and leaf traits can be observed within‐individual trees highlights the importance of within‐individual leaf trait variation in biodiversity research.

## INTRODUCTION

Trait‐based studies have become an integral part of plant ecology, as ecosystem functions are reflected in and mediated through functional traits (Laughlin, 2014; Poorter & Bongers, 2006; Violle et al., 2007). Leaf traits are particularly relevant because of their role in various ecological processes. Some of these leaf traits align with the leaf economics spectrum (LES), which characterizes a plant's return‐on‐investment strategy (Wright et al., 2004). The LES categorizes plant species along a continuum from fast‐growing/acquisitive to slow‐growing/conservative, based on their leaf trait values (Reich, 2014). One of the key indicators of the LES is specific leaf area (SLA), with high SLA values generally being associated with an acquisitive strategy. Such acquisitive leaves also tend to have higher concentrations of leaf macronutrients (Delpiano et al., 2020; Freschet et al., 2013), including high trait values for mass‐based concentration of leaf nitrogen (leaf N), magnesium (leaf Mg), phosphorus (leaf P), potassium (leaf K), calcium (leaf Ca), and sulfur (leaf S), which are linked to metabolic processes such as photosynthesis and growth (Bird et al., 1973; Jackson & Volk, 1968; Poorter et al., 2009; Terry, 1976; Tränkner et al., 2018; Wang et al., 2019). Moreover, plants with an acquisitive growth strategy tend to have higher values in chemical defense traits such as phenolics and tannins, as they typically lack structural defenses (Coley et al., 1985; Eichenberg et al., 2015). Although not all of the traits mentioned are directly involved in resource acquisition, high values of these traits are typically observed in the plants on the acquisitive side of the LES (Wright et al., 2004). Given the strong correlations between high SLA, macronutrient concentrations, and chemical defense traits, we consider them a group of correlated traits in the framework of this study (hereafter “acquisitive traits”).

By contrast, many leaf traits show high values on the conservative side of the LES (hereafter “structural traits”). For example, high values in leaf dry matter content (LDMC), cellulose, and the mass‐based carbon content (leaf C) facilitate structural integrity (Kitajima et al., 2016; Xing et al., 2021). Furthermore, plants may adjust the leaf lignin content in response to drought stress, while lignin also serves as a physical defense against pathogens (Liu et al., 2018). Finally, the leaf carbon‐to‐nitrogen ratio (CN ratio) reflects the role of N in structural compounds and metabolic processes (Xu et al., 2023) and has been reported to be positively correlated with other structural traits (Proß et al., 2021).

Leaf traits are often assessed as species mean traits (Garnier et al., 2001; McGill et al., 2006). However, within‐species leaf trait variation may contribute a substantial amount to the total variability observed within a community (Siefert et al., 2015; Violle et al., 2012). The most important drivers of within‐species leaf trait variation are ontogenetic shifts (Dayrell et al., 2018) as well as genetic variation and phenotypic plasticity (Callaway et al., 2003; Coleman et al., 1994). The latter is often caused by abiotic factors like gradients of precipitation, temperature, or elevation (Choi et al., 2019; Kühn et al., 2021; Souza et al., 2018). Within‐individual variation is rarely addressed in current studies, but existing literature indicates that it is a major contributor to overall leaf trait variation (Escribano‐Rocafort et al., 2016; Herrera, 2017; Messier et al., 2010). In forest stands, the progressive decrease in light availability from the top to the bottom of a tree's crown likely represents the most important environmental gradient (Scartazza et al., 2016). As many leaf traits are influenced by light availability (Böhnke & Bruelheide, 2013; Poorter et al., 2019), the light gradient drives the within‐individual variation in leaf trait values through adjustments in morphology and physiology (Coble & Cavaleri, 2014), particularly, in traits related to photosynthesis (Koike et al., 2001; Terashima et al., 2001; Wyka et al., 2012). Yet, changes in light conditions within the crown might affect traits idiosyncratically (Givnish, 1988). In order to maintain a net positive photosynthesis rate under low‐light conditions (i.e., at the bottom of the crown), leaves should have high values of acquisitive leaf traits and low values for structural leaf traits (Shipley et al., 2006). By contrast, under full light conditions (i.e., at the top of the crown), optimization for photosynthesis promotes thicker leaves, resulting in higher values in structural traits (Björkman, 1981).

Another factor influencing leaf traits is plant–plant interactions, which occur aboveground and belowground (Callaway et al., 2003; Davrinche & Haider, 2021; Le Bagousse‐Pinguet et al., 2013). Aboveground, light availability is a primary driver, which is itself influenced by the structure and density of the forest canopy (Pretzsch, 2014). In stands with higher species richness, spatial complementarity of tree crowns leads to more efficient crown space utilization compared to monocultures (Jucker et al., 2015; Williams et al., 2017). This results in a steeper vertical gradient of light availability, which, in turn, drives variation in light‐dependent leaf traits (Williams et al., 2020). Such biodiversity‐mediated light effects on leaf traits should be visible already at the very small scale because light availability changes drastically even across short distances (Escribano‐Rocafort et al., 2016). Moreover, in a recent study, Davrinche and Haider (2021) demonstrated that local species richness can influence multiple leaf traits, with the strongest effects often arising from a tree's closest neighbor rather than the surrounding community, hence suggesting a scale dependency of light‐mediated biodiversity effects.

Belowground interactions also influence leaf traits, albeit through different mechanisms than aboveground interactions. High species richness can enhance the availability of belowground resources through complementary resource use of different root systems (McKane et al., 2002; Turner, 2008). Belowground resource use complementarity may shift leaf trait values toward more acquisitive strategies in environments with higher species richness (Richards et al., 2010). To make use of additional belowground resources, a sufficient amount of light is necessary for photosynthesis (Freschet et al., 2015; Meziane & Shipley, 1999). Therefore, biodiversity effects on leaf traits might be stronger under full light conditions, where plants can fully utilize the enhanced resources provided in a species‐rich environment. By contrast, under low‐light conditions, the influence of biodiversity on leaf traits might decrease, as physiological constraints could outweigh the benefits of increased belowground resource availability (Dewar et al., 2012; Lloyd et al., 2010; Niinemets, 2012).

**FIGURE 1:**
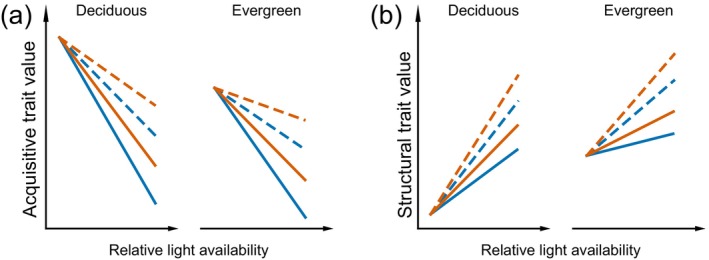
Conceptual figure of the expected interacting effects of leaf habit, light availability, and different biodiversity factors on (a) acquisitive traits, for example, leaf nitrogen content and specific leaf area; (b) structural traits, for example, leaf dry matter content and leaf carbon content. The line type indicates whether the closest neighbor of the target tree is conspecific (solid line) or heterospecific (broken line), and the line color indicates the neighborhood species richness (blue line for low, red line for high species richness). Three hypotheses are tested: H1 states that the light responsiveness of leaf traits is more pronounced in deciduous species than in evergreen species, as reflected in steeper slopes in deciduous species. H2 states that neighborhood species richness strongly affects leaf traits under full light conditions, while this effect weakens under low‐light conditions. This is visualized by all lines intersecting at low light while diverging toward full light. H3 states that the effect of the direct neighbor tree on leaf trait variation is greater than the effect of the surrounding neighborhood. This is symbolized by the broken line for heterospecific direct neighbors taking more different trait values than the solid lines for conspecific neighbors, irrespective of line color representing different species richness of the wider neighborhood.

When analyzing the light availability effect on leaf traits, it is also important to take into account the leaf habit of the tree species, as leaves of deciduous and evergreen trees strongly differ in their trait values. Deciduous trees typically have higher values in acquisitive traits and lower ones in structural traits than evergreen trees (Qin et al., 2024; Ye et al., 2022). Furthermore, there is some indication that in deciduous trees, some leaf traits respond more plastically to changing light conditions than in evergreens (Böhnke & Bruelheide, 2013; Wyka et al., 2012). In both deciduous and evergreen trees, trait responses to changes in light conditions are limited by the leaf structure (Niinemets et al., 2006; Oguchi et al., 2005). However, this limitation appears to be stronger in evergreen trees than in deciduous trees (Niinemets, 2016b). We therefore expect that leaf traits of evergreen trees will show a weaker response to the light gradient within a tree crown than that of deciduous trees. We studied leaf trait responses to light availability in the subtropical BEF‐China experiment (Biodiversity‐Ecosystem Functioning China) in forest plots differing in species richness. The aim of this study is to reveal the relationship between light availability and leaf trait values at the within‐individual level and how this relationship is mediated by local biodiversity, including the effect of the directly adjacent tree as well as the tree species richness of the surrounding neighborhood. We investigated to what extent the leaf habit of a tree might influence these interactions, assuming that leaves of evergreen trees are generally less acquisitive but possess higher values of structural traits. Further assuming that values of acquisitive traits increase with decreasing light availability (vice versa for structural traits, see Figure 1), we hypothesized that (1) the light responsiveness of leaf traits is more pronounced in deciduous species than in evergreen species. Assuming a positive effect of neighborhood species richness on acquisitive traits (vice versa for structural traits), we expected that (2) the effect of neighborhood species richness on leaf traits is more pronounced under full light conditions than under low‐light conditions. Thereby, (3) the effect of the direct neighbor tree on leaf trait variation is greater than the effect of the surrounding neighborhood.

Analyzing large datasets for leaf traits constitutes a major challenge because conventional methods often require extensive sample preparation and labor‐intensive chemical analyses (Perez‐Harguindeguy et al., 2013). Moreover, most of these methods are inherently destructive (Cornelissen et al., 2003). This is especially problematic when applied to within‐individual analyses, as samples cannot be remeasured and multiple trait measurements from the same sample cannot be combined. We therefore analyzed the leaf traits via combined visible and near‐infrared spectroscopy, which has become a well‐established method in ecological research (Trogisch et al., 2017). This method allows for nondestructive leaf‐level trait analyses (Proß et al., 2024), which helps avoid data fragmentation and enables multivariate analysis of leaf traits.

## MATERIALS AND METHODS

### Study site

The study took place in the BEF‐China experiment (Bruelheide et al., 2014), which is located in southeast China near the city of Xingangshan (Jiangxi province). The experimental site is characterized by a subtropical climate with a mean annual temperature of 16.7°C and an average annual precipitation of 1821 mm (Yang et al., 2013). The main experiment is subdivided into two experimental sites (Sites A and B). Our study took place on Site A (29.1241° N, 117.9079° E), which was established in 2009 on land that was previously covered with a forest dominated by *Pinus massoniana* lamb. The site has a total area of 18.4 ha and consists of 271 square‐shaped plots with an area of 667 m^2^ each, corresponding to the Chinese unit of 1 mu. In each plot, 400 trees were planted in a square grid pattern with a grid distance of 1.29 m between the trees. The plots consisted of monocultures and mixtures of 2, 4, 8, 16, and 24 native tree species, with species randomly assigned to positions within the grid. In the main random extinction scenario used in this study, the species of the monocultures and in the 2‐, 4‐, 8‐, and 16‐species mixtures were drawn from the same pool of 16 species, ensuring that all 16 species were equally represented at each of these diversity levels. The 24‐species mixture included an additional eight species that occurred in two other random extinction scenarios but were not part of the other mixtures included in our study. Spontaneously emerging woody plants were cut every year to maintain the experimental design.

### Sampling design

**FIGURE 2:**
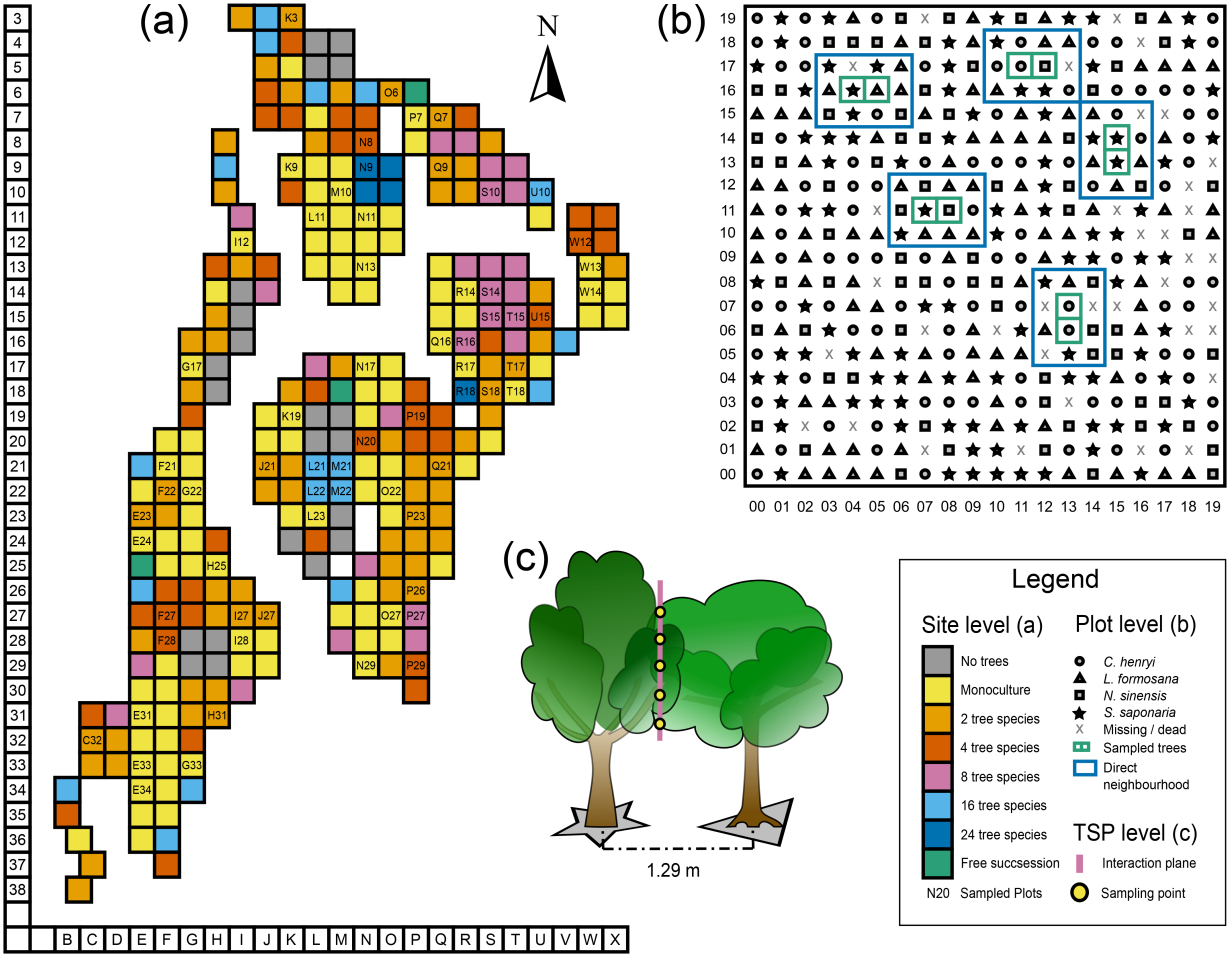
Graphical overview on the experimental design. (a) Overview of Site A of the BEF‐China main experiment near Xingangshan. The plots that were selected for sampling are marked with their *x*‐ and *y*‐coordinates (characters for *x* and numbers for *y* coordinates). The plots are color‐coded according to their initial diversity level. (b) Example for the sampling design within a plot (here Plot P19). Different species are indicated by different symbols, and dead or missing trees are indicated with *X*. Sampled trees (tree species pairs [TSPs]) are marked with green rectangles and the corresponding local neighborhood with blue rectangles. (c) Example for sampling within a TSP (here TSP 0416‐0516), showing the relationship between the focal tree and its TSP partner. Sampling took place along the vertical interaction plane of both individuals, indicated by the vertical line. Along the interaction plane, up to five sampling points (yellow points) were chosen from which at least three fully developed leaves were sampled from each tree. The illustration was created by Tobias Proß using Inkscape version 1.0.

Sampling took place in August and September 2017 in 66 plots across all plot diversity levels. We focused on the species that were present in the diversity levels up to 16 species. However, because of the high mortality of *Koelreuteria bipinnata* throughout the experimental site, we excluded it from the sampling. In total, 400 trees from the remaining 15 species (9 broad‐leaved deciduous and 6 broad‐leaved evergreen species, Appendix S1: Table S1) were selected for sampling. Trees were sampled in pairs (tree species pairs, hereafter, TSPs). TSPs consist either of two trees of the same or different species and are referred to as “Mono TSPs” and “Mixed TSPs,” respectively. While we paid attention to choose TSPs with a mostly complete neighborhood, that is, a low mortality of neighbors, the final choice of the TSPs within the plots was random. Mono TSPs were sampled throughout all plot diversity levels, and mixed TSPs were sampled in all but monocultures. The species compositions of the mixed TSPs included all possible species compositions that were present in the four‐species mixture plots (see Appendix S1: Table S2 for species pairings and Table S3 for number of samples). These species combinations also occur in the 2‐, 8‐, 16‐, and 24‐species plots. Tree species richness was assessed at the TSP level. The number of different tree species directly neighboring a TSP (with a theoretical maximum of 10 different species) was considered as a measure of its species richness (hereafter “neighborhood species richness”). The observed neighborhood species richness of the TSPs ranged from 1 to 7 for both mono and mixed TSPs. Leaf samples were taken on the side which faced the other TSP partner. The TSP partner is considered as the “direct neighbor.” Sampling was done along the vertical interaction plane of the crown between the two tree individuals of a TSP. Depending on the height of the trees, one to five sampling points were chosen in areas where crowns were intermingled. Sampling points were located between 30 cm and 8 m aboveground, with a minimum distance of 30 cm between them. From every sampling point, we aimed to collect at least three fully developed leaves from both TSP partners. Leaves were harvested without petioles, sealed in a zip‐lock bag, and stored cool until further processing in the laboratory on the same day (total sample size of 4981 leaves). For a graphical overview of the study site and the sampling design, see Figure 2.

### Light measurement

We recorded the local light availability at each sampling point using a LI‐1400 logger with a LI‐190SA quantum sensor (LI‐COR Biosciences Inc., Lincoln, NE, USA). A single reading per sampling point was used for both trees of the TSP. To account for different light conditions caused by changes in weather conditions, an identical device was located outside of the experimental site (position 29.1200° N, 117.9060° E) and fully exposed to the open sky as a reference logger. The horizontal distance between the sampling points and the reference logger ranged from 93 to 856 m. Both devices were synchronized in time (synchronization accuracy approximately 1 s) to record the photosynthetically active radiation (PAR in micromoles of photons per square meter per second) between 400 and 700 nm. The quotient of both values was used as a measure of the relative light availability at the sampling point. The method of linking two loggers was used to minimize the influence of differences in sky conditions during measurements; however, under variable sky conditions, differences might not have been fully compensated, particularly at longer distances.

### Leaf trait analysis

We acquired visible and near‐infrared spectra (Vis/NIR spectra, 350–2500 nm) from the adaxial side of each harvested leaf. Each leaf was scanned threefold with an ASD FieldSpec 4 WideRes Field Spectroradiometer (Malvern Panalytical Ltd., Almelo, the Netherlands) fitted with a “high‐bright” contact probe, including an internal 6.5 watt halogen light source. For scanning, we placed the samples between the contact probe of the FieldSpec and a black background. The three scans were averaged into single spectra in order to reduce instrument noise. A subset of 190 randomly chosen samples were analyzed for leaf traits via conventional means in the laboratory (see Appendix S1: Table S4 for reference methods). We analyzed 14 leaf traits, including LDMC, SLA, CN ratio as well as the mass‐based concentrations of leaf C, leaf N, lignin, cellulose, leaf Mg, leaf Ca, leaf K, leaf P, leaf S, phenolics, and tannin. Trait‐specific prediction models were developed via partial least squares regression (PLS regression) using “The Unscrambler X” (Version 10.1, Camo Analytics, Oslo, Norway) and “OPUS QUANT2” (Version 7.0 Bruker Optics Ltd., Billerica, MA, USA) calibration software. The models were subsequently applied to the spectral data of all leaves, yielding predicted values for each leaf trait.

### Statistical analyses

Statistical analysis was conducted using R version 4.0.3 (The R Core Team, 2020).

#### Exploratory analysis

To identify the key drivers of leaf trait variation in our dataset, we fitted a linear mixed‐effects model (“lmer” function in “lmerTest” package version 3.1‐3; Kuznetsova et al., 2017) for each leaf trait. The response variable in the models was the trait value predicted by Vis/NIR spectroscopy at the level of individual scan files. The fixed effects included light availability at the sampling point, TSP type (Mono TSP vs. Mixed TSP), and neighborhood species richness, along with all possible interactions between these variables. The model further captured how the two types of tree strategies with respect to leaf habit (deciduous and evergreen) and the different species responded differently to light availability (random slope for light across leaf habits and tree species), as well as how different species responded to differences in neighborhood species richness (random slope for neighborhood species richness across tree species).

The possible baseline differences in leaf trait values among species and leaf habits were considered (random intercept). To address the hierarchical structure of the dataset, a nested random effect was included in the models: The ID of each individual leaf was nested within the sampling point, which was nested within the individual tree, which was nested within the TSP identity, and further nested within the plot. We calculated the proportion of variance explained by each effect in the models (“calcVarPart” function in “variancePartition” package version 1.28.7; Hoffman & Schadt, 2016). To explore the relationship between the leaf traits, we calculated the pairwise correlations and performed a principal components analysis (PCA) at the sampling point level.

#### Hypothesis testing

For every leaf trait, linear mixed‐effects models were fitted (“lmer” function in “lmerTest” package version 3.1‐3; Kuznetsova et al., 2017). The response variable in the models was the trait value at each sampling point. The fixed effects included light availability at the sampling point, TSP type (Mono TSP vs. Mixed TSP), and neighborhood species richness, along with all possible interactions between these variables. Additionally, leaf habit (deciduous or evergreen) was added as the fourth fixed effect, and we allowed its interaction with light availability. This model structure captured how species may respond differently to light availability or neighborhood species richness by allowing these variables to vary across species (random slope model). Furthermore, the models accounted for possible baseline differences in leaf traits among species (random intercept model). To address the hierarchical structure of the spatial sampling, a nested random effect was included in the models: The ID of each tree individual was nested within TSP identity, which was further nested within the plot.

Since we found significant leaf habit‐by‐light interactions for many leaf traits—which suggests that the relationship between light availability and trait values differs with leaf habit—we fitted separate linear mixed‐effects models for deciduous and evergreen species, with otherwise identical model parameters. In all models, TSP type was distinguished with binary values (mono TSP = 0, mixed TSP = 1). The values of the other fixed effects (i.e., light and neighborhood richness) were scaled by dividing them by two times their SD, as this treatment makes them directly comparable to binary values (Gelman, 2008). Type 1 analysis of variance using Satterthwaite approximation for estimation of the denominator degrees of freedom was used to assess the significance of the fixed effects. All hypotheses were tested by assessing the significant effects on leaf traits in the models that were separately fitted for evergreen and deciduous species. To confirm H1, traits needed to be significantly influenced by light. To confirm H2, models should show a significant light‐by‐species richness interaction, and to confirm H3 models, it should show a significant effect of the TSP.

## RESULTS

### Leaf trait prediction models

We were able to fit a prediction model for each of the leaf traits. Model performance ranged from 0.44 for leaf K to 0.95 for SLA based on the coefficient of determination from model validation. For other performance metrics and detailed information on the prediction models, see Appendix S1: Table S5. Appendix S1: Figure S1 shows the PLS regression coefficients for every leaf trait prediction model across all wavelengths. Each leaf trait is predicted by distinct wavelengths, with some traits utilizing broader wavelength ranges than others.

### Leaf trait variation and relationship between leaf traits

**FIGURE 3:**
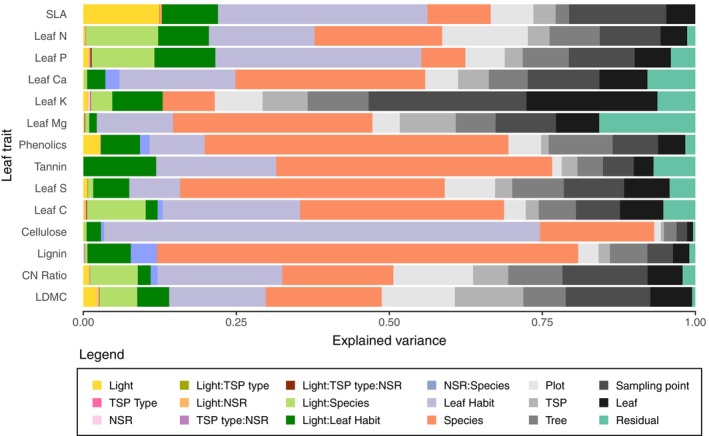
Proportions of explained variance for each leaf trait. Fixed effects include light, TSP type (tree species pair type), and NSR (neighborhood species richness), as well as all possible interactions (Light:TSP type, Light:NSR, TSP type:NSR, Light:TSP type:NSR). The model also accounts for species‐specific and leaf habit‐specific trait responses to light (Light:Species, Light:Leaf habit), as well as species‐specific leaf trait response to neighborhood species richness (NSR:Species). Random factors are nested: species within leaf habit, leaf within sampling point, sampling point within tree, tree within TSP, and TSP within plot. Residual represents unexplained variance.

All leaf traits showed strong variation between species, within‐species, and within‐individual trees (Figure 3 and Appendix S1: Figure S2). While a large portion of the variation was explained by species, species also responded differently to light, TSP type, and neighborhood richness. The unexplained variance (“residual” in Figure 3), which includes both systematic biases (i.e., model limitations) and random noise in individual predictions, is quite small compared to the other effects in the trait‐specific models.

Analysis of the leaf traits revealed that nearly all acquisitive traits were strongly correlated with one another, except for lignin and cellulose. Similarly, most structural traits exhibited strong intercorrelations (Appendix S1: Figure S3). In the PCA (Appendix S1: Figure S4), all structural traits showed positive loadings, and all acquisitive traits showed negative loadings on the first principal component. However, these traits had varying contributions to the second principal component. Only the chemical defense traits were major contributors to the third principal component.

### Leaf trait response to light availability for different leaf habits

**FIGURE 4:**
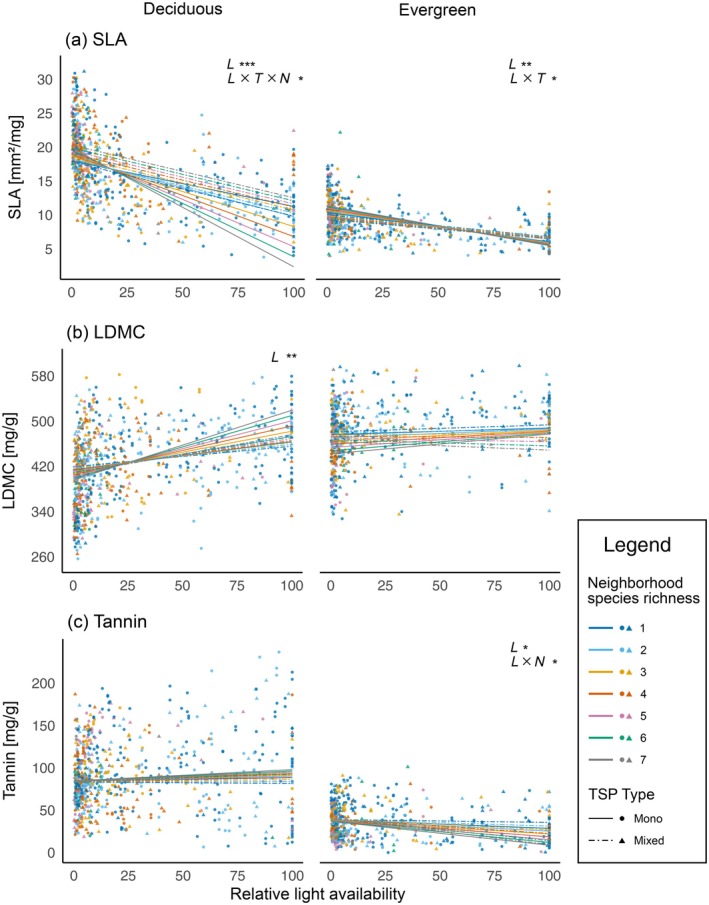
Leaf traits as functions of light availability, modified by TSP type (tree species pair type) and neighborhood species richness. Separate graphs display results for deciduous and evergreen species. Continuous lines represent monospecific TSPs; dashed lines represent mixed TSPs. Line colors correspond to different levels of tree species richness. Letters indicate significant main effects and interactions (*L* = light, *T* = TSP type, *N* = neighborhood species richness, *x* = interaction, *p < 0.05; **p < 0.01; ***p < 0.001). For the numerical results of the underlying models, see Appendix S1: Table S7. LDMC, leaf dry matter content.

Light availability strongly influenced many leaf traits. In particular, SLA, leaf K and CN ratio, phenolics, leaf C, and LDMC responded to changes in light availability (significant main effects, see Appendix S1: Table S6). However, for 7 of the 14 leaf traits, the influence of light also depended on the leaf habit (significant light‐by‐leaf habit interaction, Appendix S1: Table S6). Separate analyses for evergreen and deciduous species (Figure 4; Appendix S1: Figure S5 and Table S7) revealed that values of acquisitive traits in evergreen species, such as SLA and tannin, decreased (Figure 4a,c) while leaf N increased (Appendix S1: Figure S5a) with increasing light availability. By contrast, values of structural traits leaf C, lignin, and CN ratio decreased (Appendix S1: Figure S5h,j,k) with increasing light availability. For acquisitive traits of deciduous species, SLA, leaf P, leaf K, and leaf S decreased (Figure 4a and Appendix S1: Figure S5b,d,g) with increasing light availability. The structural trait LDMC increased with increasing light availability (Figure 4b). SLA decreased in both leaf habits, with deciduous trees showing a stronger reaction (steeper slope, Figure 4a). Finally, phenolics increased in both leaf habits, with no significant difference between the slopes (Appendix S1: Figure S5f).

### Influence of biodiversity and light availability on leaf traits

In most cases, biodiversity alone did not directly affect the leaf traits. However, biodiversity mediated the effect of light on leaf traits, as separate analyses for evergreen and deciduous species revealed multiple interactions between light and TSP type as well as between light and neighborhood species richness. For evergreen species, the observed light effect on SLA, CN ratio, and leaf N was significantly stronger in mono TSPs than in mixed TSPs (visible as steeper slopes of mono TSPs in Figure 4a and Appendix S1: Figure S5a,k). By contrast, CN ratio in deciduous trees decreased with increasing light availability in species‐rich neighborhoods, while no light effect was observed in low‐species‐richness environments (Appendix S1: Figure S5k). However, the TSP type further influenced this relationship, and mono TSPs showed an increase, while mixed TSPs showed a stronger decrease of the CN ratio in dependency on light availability (Appendix S1: Figure S5k). For evergreen species, neighborhood species richness mediated the decrease in leaf tannin content with increasing light availability: The effect of light was significantly stronger in species‐rich neighborhoods (Figure 4c).

## DISCUSSION

Overall, coupling intra‐crown leaf trait variation with detailed light measurements and tree diversity gradients demonstrates how strongly trait values adjust to local light conditions.

### Effectiveness of Vis/NIR spectroscopy for leaf trait analysis across scales

Our findings demonstrate that Vis/NIR spectroscopy can be effectively used for leaf trait analyses, especially when applied to large leaf‐to‐ecosystem datasets. While prediction accuracy varies across traits, the key limitation lies in the fact that some traits are inherently less predictable from spectral data than others. Nonetheless, this does not diminish the overall utility of the method. Some models performed exceptionally well. For example, the model for SLA achieved a very low Normalized Root Mean Square Error of 0.073 (NRMSE, which expresses the root mean square error relative to the mean of the observed values). By contrast, the model for tannin had the highest NRMSE with 0.556, indicating an average prediction error of 55.6 percent of the mean tannin value. However, our variance partitioning analysis revealed that the trait variance for tannin, as well as for most other traits, was primarily driven by species identity, leaf habit, light availability, and special variables, that is, the location of the sampled leaf, the individual tree, and the plot within the experimental site. In most cases, the residual variance was smaller than what the NRMSE of the prediction model might suggest. This implies that even for traits with moderate predictive accuracy, such as tannin, the inaccuracy of the prediction model contributes little to the unexplained variance. This is likely because the prediction errors are largely random rather than systematic, which allows the trait predictions to retain a sufficiently strong signal to reflect the underlying ecological variation.

A striking result of this study is that even the predicted values of the least accurate model (tannin) revealed significant ecological interactions when used in a linear mixed‐effects model. These results highlight a key advantage of using Vis/NIR spectroscopy for large‐scale ecological studies: Even with moderate prediction errors at the sample level, each trait prediction still retains a meaningful signal to detect ecological patterns across biological and ecological scales.

Another strength of Vis/NIR spectroscopy is that it enables the simultaneous prediction of multiple leaf traits from a single spectral measurement. This makes it a highly effective tool in large‐scale ecological studies where traditional trait measurements would be infeasible. However, concerns may arise regarding the potential nonindependence of trait predictions (Kothari & Schweiger, 2022), since all traits are derived from the same reflectance spectrum. If multiple traits were predicted from identical spectral features, this could artificially inflate trait–trait correlations. In practice, though, this concern is unwarranted. The spectral ranges used by the models to predict different traits vary considerably, and even when overlapping regions are used, the models assign different weights to spectral features (Appendix S1: Figure S1). This indicates that the predictions are based on trait‐specific spectral signatures. Therefore, while some level of correlation between the predicted traits is expected—especially for functionally related traits—the models still capture biologically meaningful and largely independent information.

### Relationship between leaf traits

All observed leaf traits showed strong alignment with the first PCA axis, with acquisitive and structural traits pointing toward opposing directions. This observation is consistent with previous studies (Delpiano et al., 2020; Domínguez et al., 2012). Hence, the first PCA axis is in our case a good representation of the LES. Additionally, traits differ in their alignment with the second PCA axis, which indicates that their variation is further influenced by other variables beyond the conservative–acquisitive trade‐off. Finally, the alignment of phenolics and tannin with the first and third PCA axis, respectively, suggests that defense traits do correlate with high values of acquisitive traits but are still influenced by another independent variable.

### Varying trait responses of evergreen and deciduous species to light

We expected that the light dependency of leaf traits was stronger in deciduous trees than in evergreens, which was the case for SLA. Furthermore, LDMC responded to changes in light conditions in deciduous, but not in evergreen, species. While the trait responses differed between evergreen and deciduous species, the overall patterns suggest that leaves of deciduous species are morphologically more adjustable to changes in light conditions, or in other words, that the response of leaves of evergreen species is more physiologically restricted, as proposed by Niinemets (2016a). The other traits (leaf P, leaf K, and leaf S) that showed a light dependency only in deciduous but not in evergreen species are all macronutrients and key elements in photosynthesis. On average, trees relocate larger proportions of phosphorus, potassium, and sulfur from their leaves before senescence compared to calcium and magnesium (Hagen‐Thorn et al., 2006). If the proportion of the recovered macronutrients can be interpreted as an indicator of their general mobility in the plant, this would be consistent with our observation that leaf P, leaf K, and leaf S respond to light conditions only in deciduous trees. A possible explanation for this pattern is that these macronutrients are essential for optimizing photosynthetic efficiency. Phosphorus plays a central role in energy metabolism, and in nucleic acid synthesis, potassium regulates stomatal conductance and enzyme activation, and sulfur is involved in amino acid and protein metabolism (Lambers et al., 2008; Terry, 1976). As light availability declines, deciduous trees may prioritize the redistribution of these elements to maximize short‐term photosynthetic gains. This greater plasticity in nutrient allocation supports faster growth and ensures efficient resource use in environments with fluctuating light conditions. The stronger adjustment of the photosynthesis‐related leaf traits in deciduous species could be a crucial mechanism to maintain plant growth under sub‐ideal conditions and hence to overall follow a faster growth strategy (Givnish, 2002). Leaves of deciduous trees represent the acquisitive side of the leaf economics spectrum (Zhao et al., 2017). As they have a shorter lifespan than leaves of evergreen trees, it is likely that the corresponding leaf traits are inherently more flexible in deciduous trees, especially in the darker sections of the crown.

However, contrary to our expectations, the light dependency of leaf N, tannin, leaf C, lignin, and CN ratio appeared to be stronger in evergreen trees. Yet, these leaf traits could be influenced by factors other than the local light availability. For lignin and tannin, a possible explanation could be found in their role as chemical defense agents.

Deciduous trees have been reported to lose more leaf area to herbivory than evergreen trees, and one contributing factor is their higher SLA (Pérez‐Harguindeguy et al., 2003; Silva et al., 2015). To counter this vulnerability, deciduous trees tend to accumulate more chemical defenses (Eichenberg et al., 2015), which is in line with our observation of higher base levels of phenolics and tannin in deciduous species. By contrast, leaves of evergreen trees are generally better protected against herbivory due to their lower SLA (Silva et al., 2015). However, we still observed an increased SLA with decreased light availability. We speculate that evergreen trees only apply chemical defenses selectively, that is, in the darker sections of the crown, where the SLA of their leaves is highest, potentially explaining our observed stronger light dependency in evergreen trees.

An alternative explanation may lie in the seasonal dynamics of carbon acquisition (Xu et al., 2024). Unlike deciduous species, evergreen trees retain their foliage year‐round, allowing them to sequester carbon dioxide outside the main growing season. Under shaded conditions, the increased SLA observed in evergreen trees indicates a strategic shift to enhance light capture. By producing thinner, more efficient leaves in low‐light conditions, evergreens can optimize carbon gain in shaded parts of the canopy while still maintaining their ability to gain carbon during cooler months. This suggests that, despite following a generally conservative strategy, evergreen species retain some degree of plasticity in leaf structure to balance light acquisition, seasonal carbon economy, and leaf longevity, much like the more acquisitive strategy seen in deciduous species.

A possible explanation for the observed increase in leaf C with decreasing light availability could be that, in leaves of evergreen trees, changes in leaf C are primarily driven by changes in lignin, which shows a similar pattern in response to light availability. The observed leaf N decrease in darker locations could be simply a dilution effect, resulting as a secondary effect from the increase in carbon compounds in these areas (Niinemets, 1997). Given these mixed results, we can only partially confirm our first hypothesis, and while light availability seems to be an important driver mainly for structural leaf traits, it has only indirect or no effects on traits associated with defense or biochemical activity.

### Influence of local biodiversity

#### Effect of neighborhood species richness in different light regimes

In most cases, leaf traits did not depend on light availability alone but also on one of the biodiversity variables (neighborhood species richness or TSP type) or a combination of both. We expected that a biodiversity effect on leaf traits would be primarily visible in full light and weaken under low‐light conditions. Tannin showed this pattern in evergreen trees, and we found lower trait values in full light conditions, especially in high species richness. A possible interpretation is that tannin allocation in forest ecosystems is a response to herbivore pressure (Hunter & Schultz, 1993), which itself depends on the tree species richness and leaf morphology (Schuldt et al., 2015; Stiegel et al., 2017), with the latter being further modified by light availability (Poorter et al., 2019). Tannin is an effective defense component against insect herbivores (Barbehenn & Constabel, 2011). However, plants might allocate resources to tannin only if necessary because they are subject to a “growth‐defense trade‐off” (Herms & Mattson, 1992), in which growth is typically prioritized over the production of secondary metabolites (Tuomi et al., 1991). In low‐light conditions, tannin content was generally higher than in full light, with no differences between levels of tree species richness. Under low‐light conditions, leaves also had higher SLA, which makes them more vulnerable to herbivory, thus making a generally higher allocation of tannin necessary. By contrast, in full‐light conditions, we found higher tannin concentrations in environments with low tree species richness than in species‐rich environments. In full light, leaves had lower SLA values, which means that they can at least partially rely on structural defense. However, in full light, tannin concentration additionally depended on the level of tree species richness, with lower tannin concentrations being observed at higher levels of tree species richness.

For the CN ratio of deciduous species, we confirmed the predicted light‐dependent biodiversity effect. In full light, leaves in species‐rich environments had a lower CN ratio than those in monocultures, while we could not detect a biodiversity effect under low‐light conditions. This is consistent with our second hypothesis. As the relative response of leaf N to biodiversity is higher than the response of leaf C, we might consider the change in CN ratio as primarily driven by changes in leaf N (Xu et al., 2023). Other studies reported an increase in leaf N with increasing biodiversity (Lang et al., 2013; Oelmann et al., 2010), possibly resulting from increasing niche partitioning in nitrogen uptake among tree species (Liu et al., 2022). However, these studies typically do not account for within‐individual trait variation (Lang et al., 2013; Liu et al., 2022; Oelmann et al., 2010). Our results suggest that a potential increase in available nitrogen due to increased species richness could result in an accumulation of nitrogen in the more sun‐exposed areas of the tree crown, where it would be utilized for photosynthesis. However, for all other leaf traits, we did not observe the predicted relationship. While many traits depended on both light and biodiversity, this relationship was not consistent.

#### Effect of the direct neighbor compared to the surrounding neighborhood

We predicted that the biodiversity influence on the light–leaf trait relationship would be greater by the direct neighbor than the species richness of the local neighborhood. When analyzing evergreen and deciduous trees together, we were not able to observe the predicted pattern. However, when analyzing leaf habits separately, we observed the predicted relationship in evergreen trees for SLA, leaf N, and CN ratio. For these traits, trees with a conspecific partner reacted stronger to changes in light conditions than those in mixed TSPs (visible as steeper slopes of the trait–light curve).

The stronger reaction of monospecific TSPs to changes in light conditions could be interpreted as a mechanism to mitigate within‐species competition between the TSP partners, which can be observed at the within‐individual level. The steeper trait–light curve indicates a greater trait variation along the light gradient, thus allowing the individuals to cover a larger trait space. A similar concept has been established among species in limiting similarity theory (MacArthur & Levins, 1967) that suggests that two species cannot coexist in the same habitat if they are too similar regarding their occupied niche. In this case, similar resource requirements would lead to greater competition. However, an increase in trait variation typically leads to a relaxation of the competition between species (Beltrán et al., 2012; Tilman, 1977), thereby enabling coexistence. Additionally, there are hints that this principle could also apply to the level of within‐species competition, that is, two individuals of the same species experience greater competition than heterospecific individuals because they are too similar regarding their resource requirements (Asay et al., 2020; File et al., 2012). By modifying their leaf traits in the presence of a conspecific competitor, both individuals can broaden their trait space. While this might not necessarily result in reduced competition, it could lead to a more efficient resource usage for both individuals. Clark (2010) demonstrated that individual‐level variation allows species to persist in competitive environments by enabling them to respond differently to environmental conditions, even when populations show no mean differences. Our observation is consistent with Proß et al. (2021) who showed that individuals of the same species show increased leaf trait variation when grown in monoculture. In our case, the observed trait shift occurs in monospecific TSPs when two individuals of the same species—that have very similar resource requirements—compete for the same aboveground resources. However, it remains unclear whether this increased intraspecific trait variation is an adaptive response that improves performance or simply a consequence of resource heterogeneity (i.e., differences in light availability or nutrient competition). Further research is needed to disentangle these possibilities. Controlled experiments would be needed that manipulate both competition intensity and resource availability to assess whether trait variation confers a competitive advantage or merely reflects environmental constraints.

Our results are further consistent with findings from Davrinche and Haider (2021) who demonstrated that leaf traits are more strongly influenced by a tree's closest neighbor than by the surrounding community. This is especially relevant because their study was conducted on the same research platform, using a similar sampling design. In species‐rich neighborhoods, they observed a shift toward a more acquisitive growth strategy for mono TSPs and attributed it to aboveground spatial niche complementarity (Davrinche & Haider, 2021). Our results indicate that apart from species richness, light availability, which itself depends on species richness, appears to be the second important driver of trait variation. This aligns with previous findings by Ellsworth and Reich (1993), who demonstrated a light‐dependent trait gradient in forest canopies, emphasizing the strong influence of light on leaf trait expression. Our findings are consistent with Williams et al. (2020) who demonstrated at the community level that leaf trait expression is mediated by light availability and biodiversity. Our results provide novel evidence that the same mechanisms are in place at the individual level.

## CONCLUSION

Our study demonstrated that within‐individual leaf trait values respond to changes in light conditions and to tree species richness of the local neighborhood. Such within‐individual leaf trait variation has rarely been recorded in this detail, yet our findings highlight the importance of this approach. Leaf traits of deciduous and evergreen species responded differently to changes in light conditions, which reflects the acquisitive or conservative growth strategy of the species. The leaf trait–light gradient within individuals was influenced by the surrounding neighborhood and the direct partner of a focal tree. This is an important distinction, as more leaf traits responded to the direct neighbor. While these findings provide insights into the potential mechanisms of species interactions, their direct role in species coexistence has yet to be demonstrated. In our case, conspecific tree pairs experienced a stronger light influence on leaf traits. The coexistence of conspecific individuals within an ecosystem is inherently hampered, as they have similar resource requirements. In this case, increased leaf trait variation could be a means of complementary resource usage and thereby enable coexistence. Additionally, our study highlights the value of Vis/NIR spectroscopy as a powerful tool for leaf trait analysis. A key advantage of this method is its ability to capture multiple leaf traits simultaneously with a single measurement, making it highly efficient for large‐scale studies. This capacity is particularly useful when applied to extensive leaf‐to‐ecosystem datasets, allowing for a more comprehensive understanding of trait variation across different spatial scales.

## AUTHOR CONTRIBUTIONS

Helge Bruelheide and Sylvia Haider conceived and designed the study. Tobias Proß conducted fieldwork and analyzed the data with support from Helge Bruelheide and Sylvia Haider. Tobias Proß wrote the first draft of the manuscript under the supervision of Sylvia Haider and Helge Bruelheide. All authors contributed to the interpretation of the results and the revision and final version of the manuscript.

## CONFLICT OF INTEREST STATEMENT

The authors declare no conflicts of interest.

## Supporting information


Appendix S1.

## Data Availability

Data (Proß et al., 2025) are available on Zenodo at https://doi.org/10.5281/zenodo.15584525.
