# Supplementary material for: Within‐individual leaf trait response to local light availability and biodiversity in a subtropical forest experiment
Source: Ecology. 2025 Jul 18;106(7):e70160. doi: 10.1002/ecy.70160 (PMC12272144; doi:10.1002/ecy.70160)
Supplement: Supplementary file 1 — Appendix S1. [file ECY-106-e70160-s001.pdf]

Appendix S1

Supplementary information

Journal: Ecology

Title: Within-individual leaf trait response to local light availability and biodiversity in a subtropical forest experiment

Authors: Tobias Proß, Helge Bruelheide, Sylvia Haider

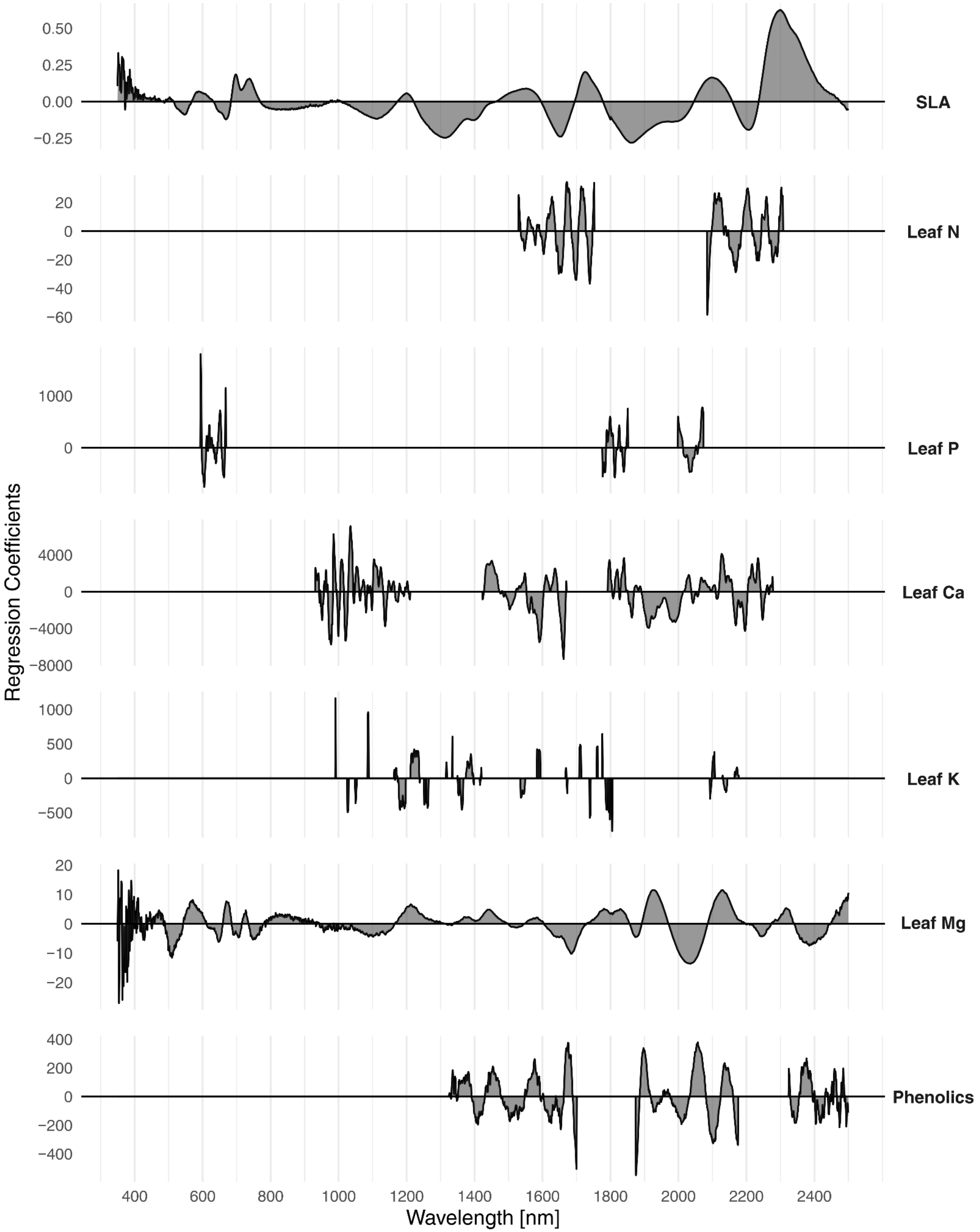

Figure S1. Regression coefficients for the highest rank of the leaf trait prediction models. For model ranks see Table S5.

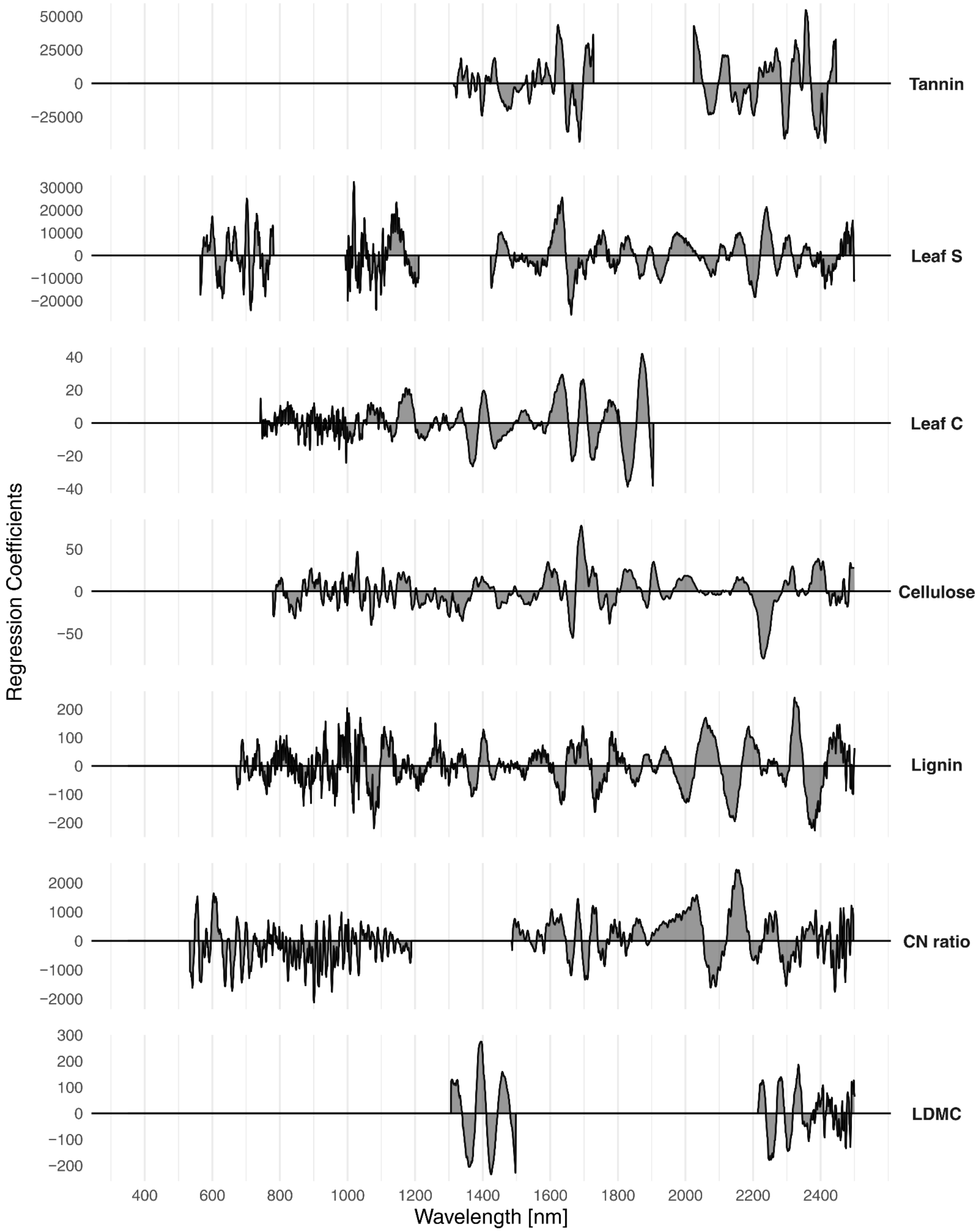

Figure S1 (cont.). Regression coefficients for the highest rank of the leaf trait prediction models. For model ranks see Table S5.

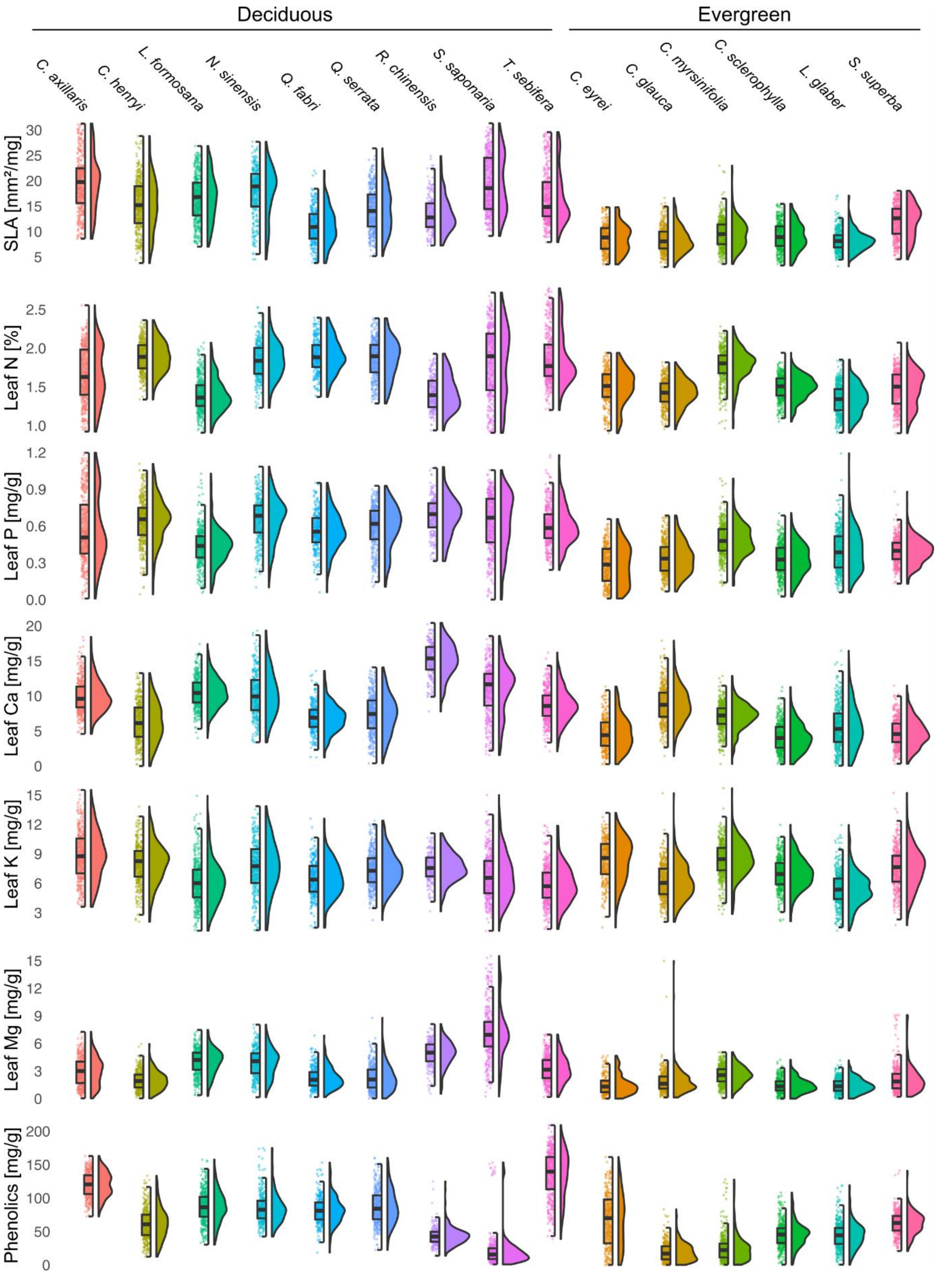

Figure S2. Species-wise overview of leaf traits. Each datapoint represents a separate leaf. For full species names, see Table S1.

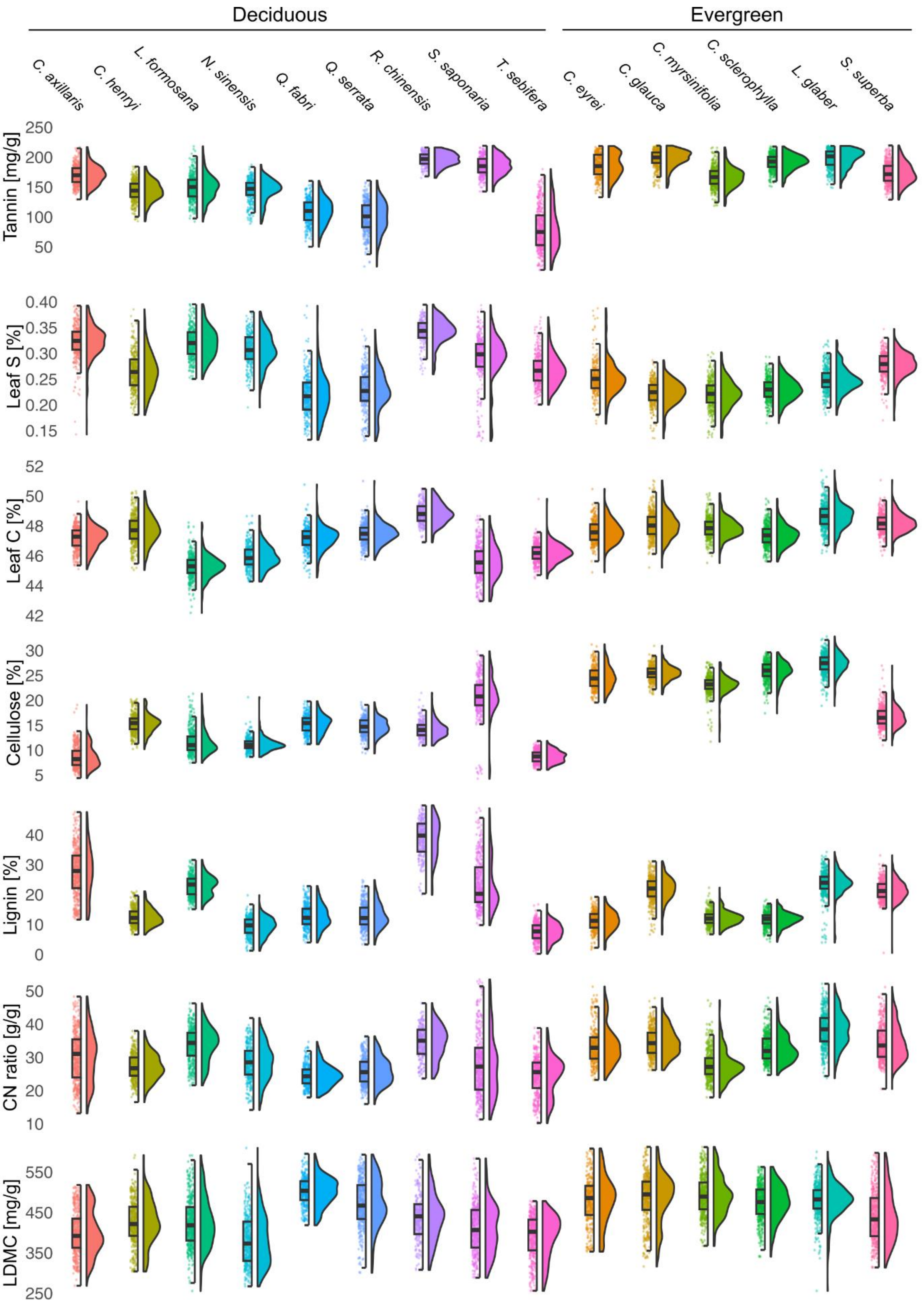

Figure S2 (cont). Species-wise overview of leaf traits. Each datapoint represents a separate leaf. For full species names, see Table S1.

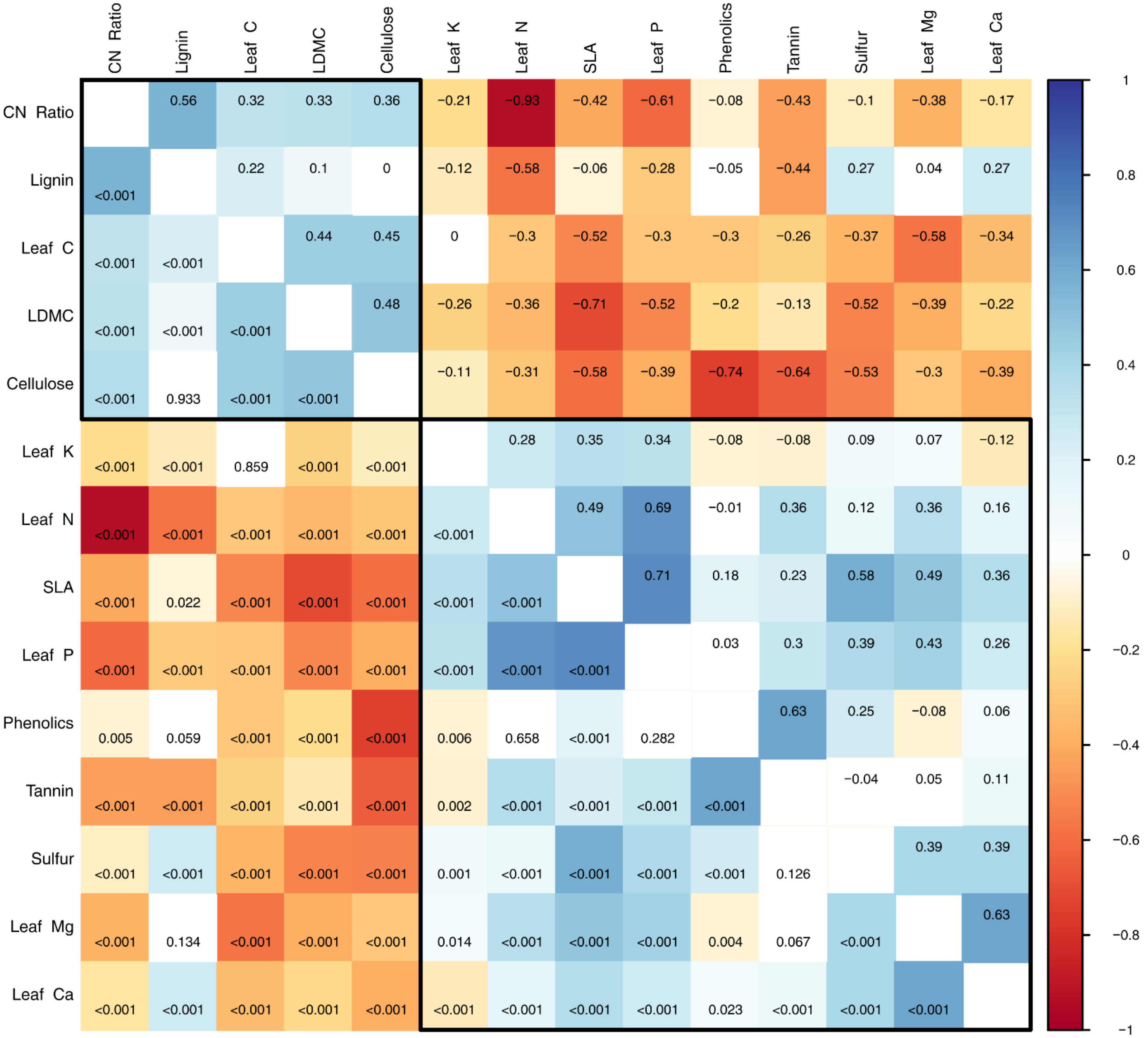

Figure S3. Correlation between all measured leaf traits. Each of the possible trait pairings is present twice: Top values and colors indicate magnitude and direction of the pairwise correlation between leaf traits. Bottom values indicate p-values to identify significant correlations. Calculations are based on Pearson's product moment correlation coefficient. Black boxes indicate the two most similar groups of traits, based on hierarchical cluster analysis.

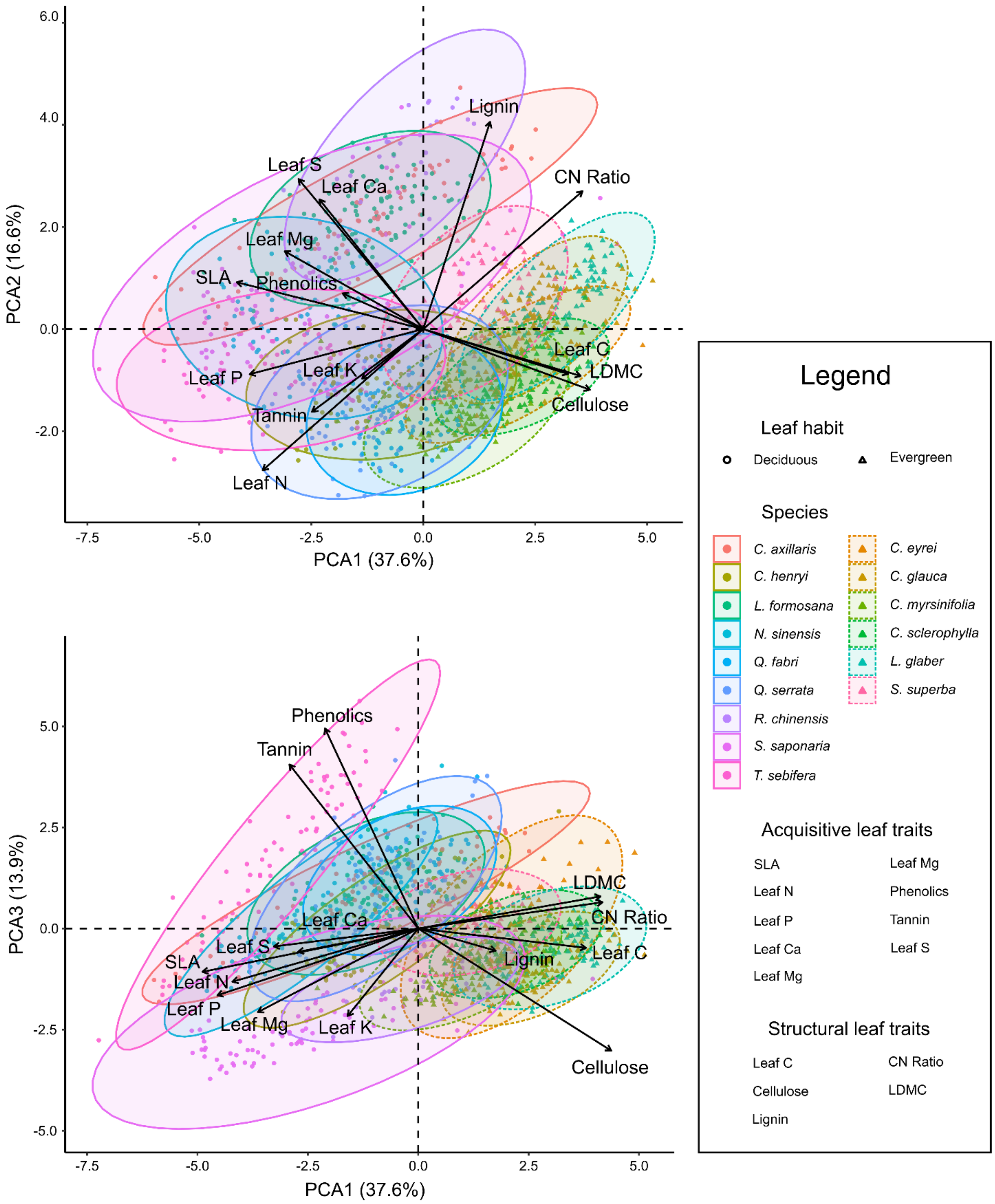

Figure S4. Principal component analysis of all measured leaf traits. Top: first vs. second axis. Bottom: first vs. third axis. Each datapoint represents a separate sampling point. Ellipses contain 95% of the datapoints of each species. Colors indicate different species, shapes and ellipse line types indicate their leaf habit. For full species names, see Table S1.

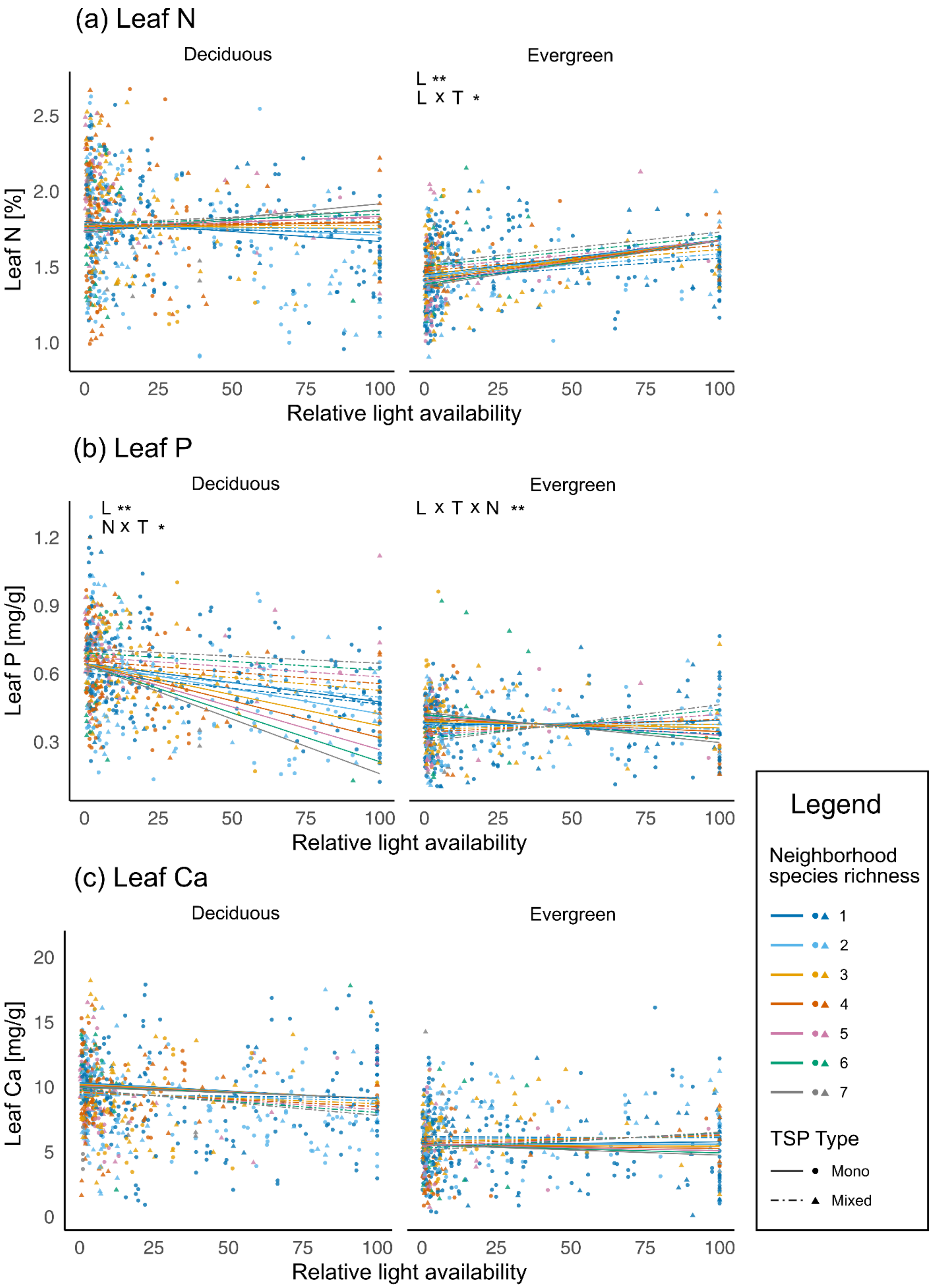

Figure S5 (a-c). Leaf traits as functions of light availability, modified by TSP type (Tree species pair type) and neighborhood species richness. Separate graphs for deciduous and evergreen species. The continuous lines indicate monospecific TSPs, dashed lines indicate mixed TSPs. Different colors indicate different levels of neighborhood species richness. Letters indicate significant main effects and interactions (L = Light, T = TSP type, N = Neighborhood species richness, x = interaction, * $p < 0.05$, ** $p < 0.01$, *** $p < 0.001$). For the numeric results of the underlaying models see Table S7.

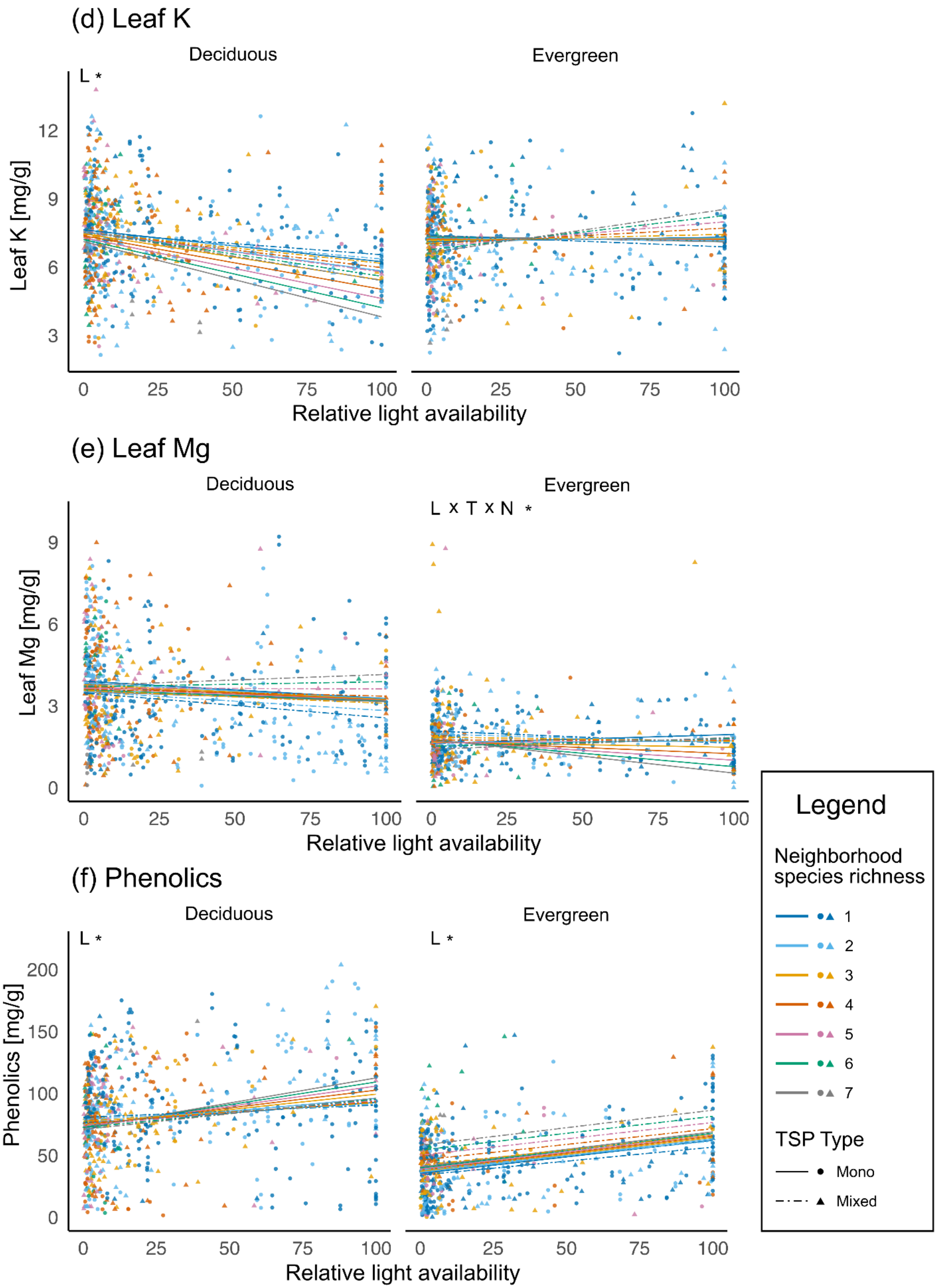

Figure S5 (d-f). Leaf traits as functions of light availability, modified by TSP type (Tree species pair type) and neighborhood species richness. Separate graphs for deciduous and evergreen species. The continuous lines indicate monospecific TSPs, dashed lines indicate mixed TSPs. Different colors indicate different levels of neighborhood species richness. Letters indicate significant main effects and interactions (L = Light, T = TSP type, N = Neighborhood species richness, x = interaction, * $p < 0.05$, ** $p < 0.01$, *** $p < 0.001$). For the numeric results of the underlaying models see Table S7.

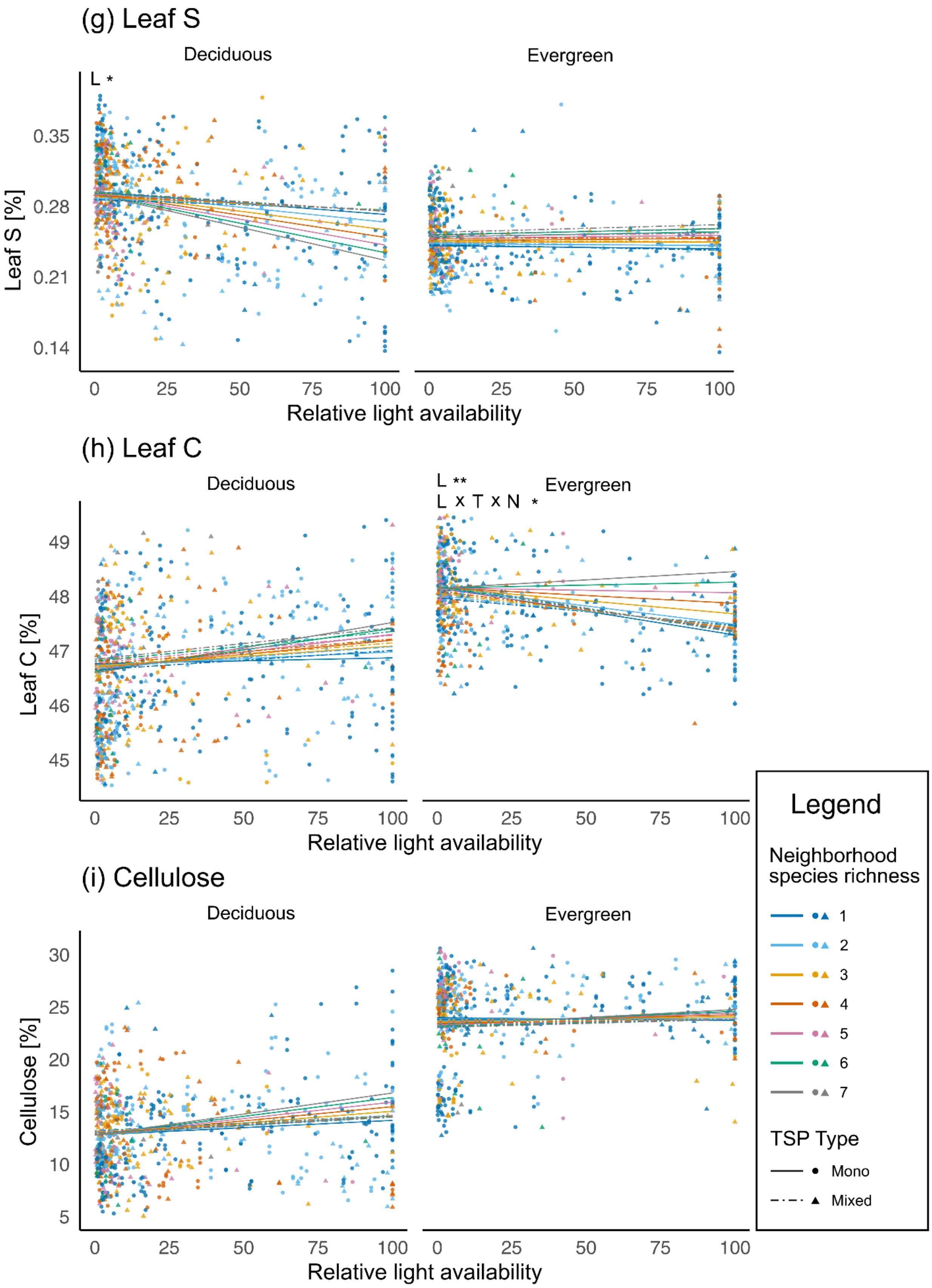

Figure S5 (g-i). Leaf traits as functions of light availability, modified by TSP type (Tree species pair type) and neighborhood species richness. Separate graphs for deciduous and evergreen species. The continuous lines indicate monospecific TSPs, dashed lines indicate mixed TSPs. Different colors indicate different levels of neighborhood species richness. Letters indicate significant main effects and interactions (L = Light, T = TSP type, N = Neighborhood species richness, x = interaction, * $p < 0.05$, ** $p < 0.01$, *** $p < 0.001$). For the numeric results of the underlaying models see Table S7.

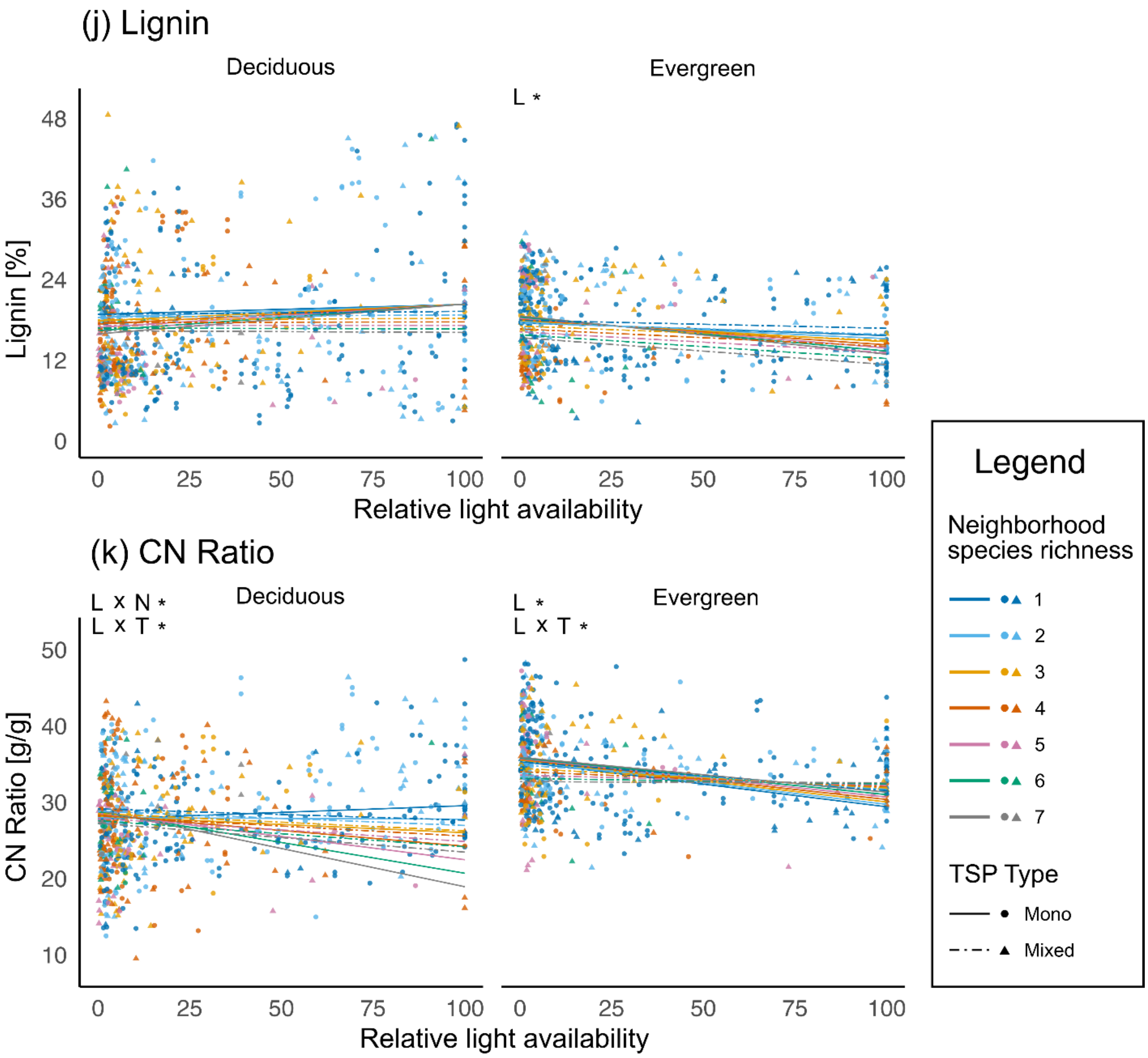

Figure S5 (j-k). Leaf traits as functions of light availability, modified by TSP type (Tree species pair type) and neighborhood species richness. Separate graphs for deciduous and evergreen species. The continuous lines indicate monospecific TSPs, dashed lines indicate mixed TSPs. Different colors indicate different levels of neighborhood species richness. Letters indicate significant main effects and interactions (L = Light, T = TSP type, N = Neighborhood species richness, x = interaction, * $p < 0.05$, ** $p < 0.01$, *** $p < 0.001$). For the numeric results of the underlaying models see Table S7.

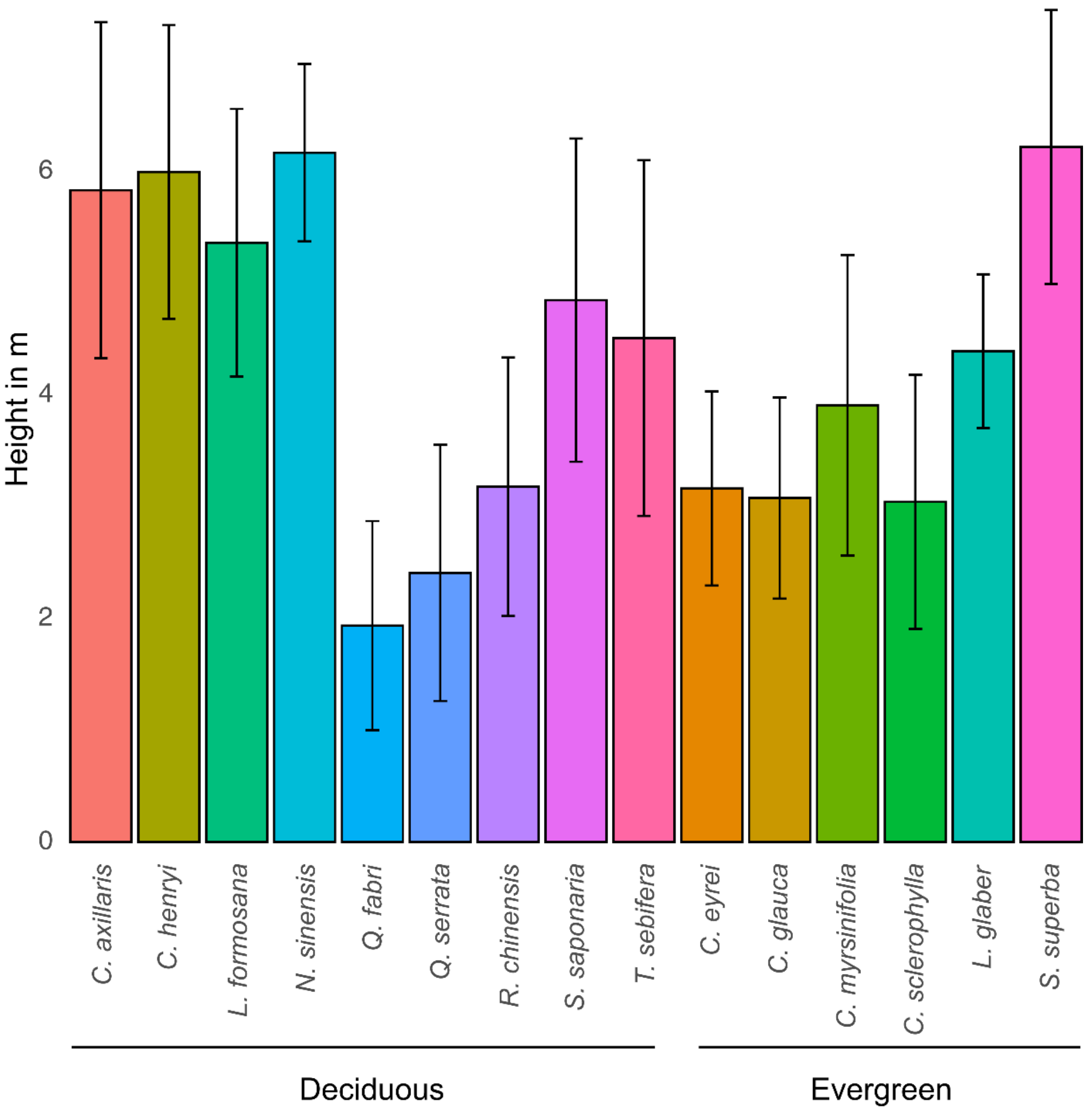

Figure S6. Approximate height of the sampled trees. Colored bars indicate the average height per species, the error bars indicate the standard deviation. For full species names, see Table S1.

Table S1. Species list. Note that some of the species have been described under multiple names or have undergone taxonomic revisions, including reassignments to different genera. While alternative classifications may exist in other taxonomic sources, the species names throughout this publication follow the nomenclature and classification used in the Flora of China (Brach and Song 2006; eFloras 2008).

Species	Abbreviation	Family	Leaf habit
Choerospondias axillaris (ROXB.) B.L.BURTT & A.W.HILL	*C. axillaris*	Anacardiaceae	Deciduous
Castanea henryi (SKAN) REHDER & E.H.WILSON	*C. henryi*	Fagaceae	Deciduous
Liquidambar formosana HANCE	*L. formosana*	Altingiaceae	Deciduous
Nyssa sinensis OLIVER	*N. sinensis*	Nyssaceae	Deciduous
Quercus fabri HANCE	*Q. fabri*	Fagaceae	Deciduous
Quercus serrata MURRAY	*Q. serrata*	Fagaceae	Deciduous
Rhus chinensis MILLER	*R. chinensis*	Anacardiaceae	Deciduous
Sapindus saponaria GAERTNER	*S. saponaria*	Sapindaceae	Deciduous
Triadica sebifera (LINNAEUS) SMALL	*T. sebifera*	Euphorbiaceae	Deciduous
Castanopsis eyrei (CHAMPION EX BENTHAM) HUTCHINSON	*C. eyrei*	Fagaceae	Evergreen
Cyclobalanopsis glauca (THUNBERG) OERSTED	*C. glauca*	Fagaceae	Evergreen
Cyclobalanopsis myrsinifolia (BLUME) OERSTED	*C. myrsinifolia*	Fagaceae	Evergreen
Castanopsis sclerophylla (LINDLEY & PAXTON) SCHOTTKY	*C. sclerophylla*	Fagaceae	Evergreen
Lithocarpus glaber (THUNBERG) NAKAI	*L. glaber*	Fagaceae	Evergreen
Schima superba GARDNER & CHAMPION	*S. superba*	Theaceae	Evergreen

Table S2. Number of sampled trees by species. The selection of the species pairings is based on the species combinations that occur in 4-species mixture plots. The # Trees refers to the first species of the pair and indicates how many individuals of this species are paired with the corresponding species. For full species names, see table S1.

Species pairing	# Trees	Species pairing	# Trees	Species pairing	# Trees
C. axillaris - C. axillaris	18	*C. sclerophylla - C. sclerophylla*	18	*Q. serrata - Q. serrata*	18
C. axillaris - C. sclerophylla	3	*C. sclerophylla - C. axillaris*	3	*Q. serrata - C. axillaris*	3
C. axillaris - Q. serrata	3	*C. sclerophylla - Q. serrata*	6	*Q. serrata - C. sclerophylla*	6
C. axillaris - T. sebifera	6	*C. sclerophylla - T. sebifera*	3	*Q. serrata - T. sebifera*	2
C. eyrei - C. eyrei	8	*L. formosana - L. formosana*	18	*R. chinensis - R. chinensis*	6
C. eyrei - C. myrsinifolia	4	*L. formosana - C. henryi*	3	*R. chinensis - C. glauca*	3
C. eyrei - L. glaber	4	*L. formosana - N. sinensis*	3	*R. chinensis - K. bipinnata*	1
C. eyrei - K. bipinnata	0	*L. formosana - S. saponaria*	6	*R. chinensis - S. superba*	1
C. glauca - C. glauca	18	*L. glaber - L. glaber*	18	*S. saponaria - S. saponaria*	18
C. glauca - Q. fabri	6	*L. glaber - C. eyrei*	4	*S. saponaria - C. henryi*	3
C. glauca - R. chinensis	3	*L. glaber - C. myrsinifolia*	3	*S. saponaria - L. formosana*	6
C. glauca - S. superba	3	*L. glaber – K. bipinnata*	3	*S. saponaria - N. sinensis*	3
C. henryi - C. henryi	18	*N. sinensis - N. sinensis*	16	*T. sebifera - T. sebifera*	16
C. henryi - L. formosana	3	*N. sinensis - C. henryi*	7	*T. sebifera - C. axillaris*	6
C. henryi - N. sinensis	7	*N. sinensis - L. formosana*	3	*T. sebifera - C. sclerophylla*	3
C. henryi - S. saponaria	3	*N. sinensis - S. saponaria*	3	*T. sebifera - Q. serrata*	2
C. myrsinifolia - C. myrsinifolia	18	*Q. fabri - Q. fabri*	18	*S. superba - S. superba*	18
C. myrsinifolia - C. eyrei	4	*Q. fabri - C. glauca*	6	*S. superba - C. glauca*	3
C. myrsinifolia - K. bipinnata	3	*Q. fabri - K. bipinnata*	1	*S. superba - Q. fabri*	2
C. myrsinifolia - L. glaber	3	*Q. fabri - S. superba*	2	*S. superba - R. chinensis*	1

Table S3. Number of samples by species and neighborhood species richness level (NSR). For full species names, see Table S1.

Species	Samples per NSR level (Mono)							Samples per NSR level (Mixed)						
	**1**	**2**	**3**	**4**	**5**	**6**	**7**	**1**	**2**	**3**	**4**	**5**	**6**	**7**
C. axillaris	20	11	0	16	0	0	0	3	10	4	10	5	3	0
C. henryi	23	8	0	0	0	0	0	12	10	0	4	4	4	0
L. formosana	24	27	7	0	7	0	0	6	11	18	0	3	0	3
N. sinensis	23	16	8	0	5	4	0	0	13	6	10	2	2	3
Q. fabri	23	0	6	7	0	0	0	13	9	3	3	4	3	0
Q. serrata	31	16	0	24	0	0	0	0	10	7	13	3	2	0
R. chinensis	22	23	7	17	0	0	0	3	8	8	11	0	11	0
S. saponaria	40	8	7	0	8	0	0	7	15	5	0	4	8	0
T. sebifera	16	15	6	7	0	0	0	0	17	4	6	5	5	4
C. eyrei	28	8	6	0	12	0	0	2	11	10	0	0	0	0
C. glauca	29	13	5	7	0	0	5	0	8	4	14	2	0	0
C. myrsinifolia	10	0	0	0	0	0	0	0	0	8	0	4	0	0
C. sclerophylla	14	17	0	12	0	3	0	3	4	10	9	8	0	0
L. glaber	29	9	0	5	4	0	0	2	10	7	7	10	0	0
S. superba	42	8	7	0	8	0	0	4	4	14	0	0	0	4

Table S4. Reference methods for leaf trait analysis. The samples were rehydrated and weighted freshly and scanned with a flatbed scanner on the day of harvest. The samples were dried for 72 hours at 60 °C and weighed again. LDMC was calculated as dry weight per unit fresh weight and SLA was calculated as leaf area per unit dry mass. As a preparation for other analyses, calibration samples were ground to a homogenous powder using a Retsch MM400 ball mill. Ground samples were further treated via nitric acid digestion and diluted with a Cs/Ln buffer solution. Reference samples and phenolics and tannin data for calibration were kindly provided by Eichenberg et al. (2014).

Leaf trait	Method, instrument or software	Reference
LDMC	Weighting of fresh and dried leaves with precision scale (Sartorius, Göttingen, Germany)	Pérez-Harguindeguy et al. 2016
SLA	Leaf area calculation with WinFOLIA (v Pro 2004a Regent Instruments)	Pérez-Harguindeguy et al. 2016
Lignin, Cellulose	Van Soest analysis using a ANKOM200 Fiber Analyzer (ANKOM Technology, Macedon NY, USA)	Van Soest et al. 1991
Leaf C, Leaf N, Leaf S, CN ratio	Gas chromatography using a vario EL cube (Elementar, Hanau, Germany)	-
Leaf Ca, Leaf Mg, Leaf K,	Atomic absorption spectrometry using a SpectrAA 300 (Analytik Jena GmbH, Jena, Germany)	Schinkel 1984
Leaf P	Molybdenum blue assay using a GENESYS 140 Spectrophotometer (Thermo Fisher Scientific Inc., Waltham MA, USA)	Murphy and Riley 1958
Phenolics	Modified Prussian-blue assay	Graham 1992; Price and Butler 1977; Eichenberg et al. 2014
Tannin	Protein-precipitation based radial diffusion assay	Hagerman 1987; Eichenberg et al. 2014

Table S5. Details of the leaf trait prediction models. A separate model was created for each leaf trait. The dataset, consisting of 190 samples, was divided into two subsets: a calibration (training) set and a validation (test) set. Each subset contained 95 samples, ensuring that all species were represented in both sets. For most models, spectral data underwent pre-treatment, with the optimal method chosen empirically for each leaf trait. Several pre-treatment methods were tested, including standard normal variate (SNV), multiplicative scatter correction (MSC), first and second derivative using Savitzky-Golay algorithm (1D, 2D), min-max normalization (MMN) as well as a combination of these (+), or no data pre-treatment (RAW). Relevant spectral regions were selected empirically to optimize predictive performance. The models were trained using the calibration dataset and subsequently validated using the independent test dataset. The final model for each trait was selected based on the lowest root mean square error of prediction (RMSEP). Additional performance metrics are reported, including normalized root mean square error (NRMSE, i.e. RMSEP divided by mean trait value), coefficient of determination (R^2), ratio of standard deviation of the sample to standard error of the prediction (RPD). The model rank indicates the number of latent factors in the regression. For regression coefficients figure S1.

Leaf trait	Mean trait value	RMSEP	NRMSE	R^2	RPD	Rank	Data pre-treatment	Spectral regions in nm
SLA	13.23 mm²/mg	0.96	0.0726	0.95	4.51	6	SNV	350-2500
Leaf N	1.64 %	0.12	0.0731	0.85	2.56	15	MSC	1530-1752;<br>2085-2307
Leaf P	0.49 mg/g	0.15	0.3042	0.63	1.65	13	1D	991-1855;<br>2069-2177
Leaf Ca	7.69 mg/g	1.81	0.2354	0.79	2.19	13	1D	924-1210; 1424-1670;<br>1793-2285
Leaf K	7.07 mg/g	2.13	0.3012	0.44	1.33	15	MSC	991-1099
Leaf Mg	2.71 mg/g	0.76	0.2801	0.88	2.90	15	RAW	350-2500
Phenolics	65.76 mg/g	23.00	0.3498	0.75	1.98	13	MMN	1325-1700; 1875-<br>2175; 2325-2500
Tannin	65.88 mg/g	36.60	0.5556	0.66	1.73	7	1D + MSC	1315-1727;<br>2025-2445
Leaf S	0.26 mg/g	0.06	0.2266	0.61	1.59	19	MSC	565-780; 995-1210;<br>1425-2500
Leaf C	47.23 %	0.90	0.0191	0.74	1.97	11	MSC	743-1904
Cellulose	18.00 %	2.38	0.1323	0.87	2.77	9	1D + SNV	780-2500
Lignin	17.37 %	3.04	0.1750	0.87	2.79	24	RAW	671-2500
CN ratio	30.67 g/g	2.04	0.0665	0.89	3.01	15	1D	530-1188;<br>1487-2500
LDMC	446.77 mg/g	18.04	0.0404	0.91	3.38	9	SNV	1307-1497;<br>2215-2500

Table S6. Results of the type I ANOVA of linear mixed-effects models including both leaf habits (n=1314) for each leaf trait. Models were fitted with the trait values as response variables and light availability, TSP type (Tree species pair type), neighborhood species richness and leaf habit as fixed effects. Colons indicate interactions between fixed effects, significant results ($p < 0.05$) are highlighted in bold.

Trait	Predictors	*F*-value	*p*-value
SLA	Light availability	93.61	**< 0.001**
	TSP type	2.79	0.098
	Neighborhood species richness	12.63	**0.001**
	Leaf habit	45.37	**< 0.001**
	Light availability : TSP type	1.96	0.162
	Light availability : Neighborhood species richness	6.35	**0.012**
	TSP type : Neighborhood species richness	0.01	0.913
	Light availability : Leaf habit	12.28	**0.005**
	Light availability : TSP type : Neighborhood species richness	2.73	0.099
Leaf N	Light availability	2.03	0.177
	TSP type	0.39	0.532
	Neighborhood species richness	2.93	0.090
	Leaf habit	7.37	**0.017**
	Light availability : TSP type	0.03	0.872
	Light availability : Neighborhood species richness	0.72	0.398
	TSP type : Neighborhood species richness	0.51	0.477
	Light availability : Leaf habit	9.65	**0.009**
	Light availability : TSP type : Neighborhood species richness	0.22	0.637
Leaf P	Light availability	4.47	0.052
	TSP type	0.43	0.515
	Neighborhood species richness	3.10	0.088
	Leaf habit	31.49	**< 0.001**
	Light availability : TSP type	0.96	0.327
	Light availability : Neighborhood species richness	1.77	0.184
	TSP type : Neighborhood species richness	0.49	0.487
	Light availability : Leaf habit	6.05	**0.028**
	Light availability : TSP type : Neighborhood species richness	11.24	**< 0.001**
Leaf Ca	Light availability	0.45	0.512
	TSP type	0.06	0.812
	Neighborhood species richness	2.90	0.095
	Leaf habit	11.33	**0.005**
	Light availability : TSP type	0.21	0.646
	Light availability : Neighborhood species richness	1.15	0.285
	TSP type : Neighborhood species richness	0.26	0.609
	Light availability : Leaf habit	2.38	0.147
	Light availability : TSP type : Neighborhood species richness	0.14	0.708
Leaf K	Light availability	4.94	**0.043**
	TSP type	0.28	0.597
	Neighborhood species richness	0.94	0.334
	Leaf habit	0.32	0.578
	Light availability : TSP type	0.00	0.952

Trait	Predictors	***F*-value**	***p*-value**
	Light availability : Neighborhood species richness	0.76	0.385
	TSP type : Neighborhood species richness	0.08	0.777
	Light availability : Leaf habit	6.10	**0.028**
	Light availability : TSP type : Neighborhood species richness	1.38	0.241
Leaf Mg	Light availability	0.90	0.354
	TSP type	0.00	0.995
	Neighborhood species richness	2.54	0.115
	Leaf habit	8.23	**0.013**
	Light availability : TSP type	0.81	0.369
	Light availability : Neighborhood species richness	0.21	0.648
	TSP type : Neighborhood species richness	0.06	0.808
	Light availability : Leaf habit	3.37	0.087
	Light availability : TSP type : Neighborhood species richness	6.44	**0.011**
Phenolics	Light availability	20.81	< **0.001**
	TSP type	2.74	0.100
	Neighborhood species richness	3.78	0.061
	Leaf habit	3.57	0.081
	Light availability : TSP type	0.80	0.370
	Light availability : Neighborhood species richness	0.35	0.555
	TSP type : Neighborhood species richness	0.17	0.680
	Light availability : Leaf habit	0.49	0.496
	Light availability : TSP type : Neighborhood species richness	0.00	0.951
Tannin	Light availability	0.00	0.967
	TSP type	0.12	0.733
	Neighborhood species richness	5.23	**0.029**
	Leaf habit	8.42	**0.013**
	Light availability : TSP type	0.00	1.000
	Light availability : Neighborhood species richness	0.55	0.459
	TSP type : Neighborhood species richness	0.11	0.742
	Light availability : Leaf habit	1.30	0.276
	Light availability : TSP type : Neighborhood species richness	0.09	0.770
Leaf S	Light availability	3.58	0.079
	TSP type	0.63	0.430
	Neighborhood species richness	3.49	0.067
	Leaf habit	4.64	0.051
	Light availability : TSP type	1.39	0.238
	Light availability : Neighborhood species richness	0.04	0.852
	TSP type : Neighborhood species richness	0.00	0.993
	Light availability : Leaf habit	3.54	0.084
	Light availability : TSP type : Neighborhood species richness	0.84	0.360
Leaf C	Light availability	6.44	**0.023**
	TSP type	0.46	0.498
	Neighborhood species richness	2.01	0.164
	Leaf habit	6.83	**0.021**
	Light availability : TSP type	0.65	0.421

Trait	Predictors	*F*-value	*p*-value
	Light availability : Neighborhood species richness	10.10	**0.002**
	TSP type : Neighborhood species richness	1.75	0.187
	Light availability : Leaf habit	22.03	< **0.001**
	Light availability : TSP type : Neighborhood species richness	4.31	**0.038**
Cellulose	Light availability	0.71	0.414
	TSP type	1.61	0.207
	Neighborhood species richness	19.90	< **0.001**
	Leaf habit	27.43	< **0.001**
	Light availability : TSP type	0.03	0.873
	Light availability : Neighborhood species richness	5.62	**0.018**
	TSP type : Neighborhood species richness	0.02	0.884
	Light availability : Leaf habit	1.92	0.191
	Light availability : TSP type : Neighborhood species richness	1.50	0.220
Lignin	Light availability	0.01	0.938
	TSP type	2.15	0.146
	Neighborhood species richness	0.49	0.487
	Leaf habit	0.03	0.865
	Light availability : TSP type	0.55	0.457
	Light availability : Neighborhood species richness	0.85	0.357
	TSP type : Neighborhood species richness	0.23	0.629
	Light availability : Leaf habit	2.71	0.124
	Light availability : TSP type : Neighborhood species richness	0.60	0.441
CN ratio	Light availability	14.33	**0.001**
	TSP type	0.46	0.499
	Neighborhood species richness	3.71	0.057
	Leaf habit	8.70	**0.010**
	Light availability : TSP type	0.26	0.607
	Light availability : Neighborhood species richness	0.28	0.594
	TSP type : Neighborhood species richness	0.56	0.458
	Light availability : Leaf habit	15.46	**0.002**
	Light availability : TSP type : Neighborhood species richness	0.00	0.997
LDMC	Light availability	16.36	**0.001**
	TSP type	0.04	0.844
	Neighborhood species richness	4.99	**0.027**
	Leaf habit	7.26	**0.018**
	Light availability : TSP type	1.54	0.215
	Light availability : Neighborhood species richness	2.18	0.141
	TSP type : Neighborhood species richness	0.16	0.688
	Light availability : Leaf habit	6.65	**0.025**
	Light availability : TSP type : Neighborhood species richness	1.25	0.265

Table S7. Results of the type I ANOVA. Linear mixed-effects models were fitted for each leaf trait and for both deciduous (n=725) and evergreen (n=589) trees separately. The trait values are response variables and light availability, TSP type (Tree species pair type) and neighborhood species richness (NSR) are fixed effects. Colons indicate interactions between fixed effects, significant results ($p < 0.05$) are highlighted in bold.

		Deciduous species		Evergreen species	
**Trait**	**Predictor**	***F*-value**	***p*-value**	***F*-value**	***p*-value**
SLA	Light	59.67	< **0.001**	26.13	**0.004**
	TSP Type	0.90	0.347	1.05	0.312
	NSR	0.80	0.387	0.00	0.955
	Light : TSP Type	0.40	0.530	6.26	**0.013**
	Light : NSR	3.43	0.065	0.19	0.663
	TSP Type : NSR	1.61	0.207	1.48	0.228
	Light : TSP Type : NSR	4.07	**0.044**	0.49	0.483
Leaf N	Light	0.79	0.394	17.77	**0.008**
	TSP Type	0.14	0.714	0.07	0.795
	NSR	0.01	0.917	0.18	0.675
	Light : TSP Type	2.99	0.085	6.19	**0.013**
	Light : NSR	2.61	0.107	0.15	0.699
	TSP Type : NSR	0.30	0.584	2.46	0.122
	Light : TSP Type : NSR	0.48	0.490	0.00	0.988
Leaf P	Light	10.99	**0.008**	0.00	0.952
	TSP Type	1.60	0.210	1.22	0.275
	NSR	0.25	0.625	0.04	0.857
	Light : TSP Type	1.68	0.196	0.02	0.900
	Light : NSR	0.78	0.377	0.00	0.979
	TSP Type : NSR	5.52	**0.021**	1.12	0.294
	Light : TSP Type : NSR	3.23	0.073	7.15	**0.008**
Leaf Ca	Light	2.48	0.144	0.32	0.595
	TSP Type	3.22	0.076	1.16	0.287
	NSR	0.01	0.909	0.42	0.530
	Light : TSP Type	0.14	0.710	0.11	0.745
	Light : NSR	0.09	0.759	0.00	0.995
	TSP Type : NSR	0.12	0.732	0.32	0.575
	Light : TSP Type : NSR	0.46	0.500	0.99	0.320
Leaf K	Light	9.17	**0.013**	0.04	0.855
	TSP Type	0.09	0.760	0.01	0.916
	NSR	0.92	0.340	0.03	0.866
	Light : TSP Type	0.01	0.931	0.05	0.828
	Light : NSR	1.14	0.286	0.46	0.499
	TSP Type : NSR	0.62	0.432	0.27	0.605
	Light : TSP Type : NSR	0.08	0.783	1.87	0.172
Leaf Mg	Light	3.51	0.079	0.08	0.782
	TSP Type	1.10	0.296	0.72	0.400
	NSR	0.00	0.977	0.92	0.353
	Light : TSP Type	0.01	0.944	2.84	0.093
	Light : NSR	1.00	0.317	1.23	0.269

Trait	Predictor	Deciduous species *F*-value	Deciduous species *p*-value	Evergreen species *F*-value	Evergreen species *p*-value
	TSP Type : NSR	1.08	0.301	0.58	0.449
	Light : TSP Type : NSR	0.33	0.567	5.04	**0.025**
Phenolics	Light	6.15	**0.034**	16.05	**0.010**
	TSP Type	0.08	0.778	2.17	0.146
	NSR	0.26	0.615	3.83	0.095
	Light : TSP Type	0.19	0.666	1.94	0.164
	Light : NSR	1.62	0.204	0.00	0.981
	TSP Type : NSR	0.47	0.493	2.69	0.105
	Light : TSP Type : NSR	0.08	0.774	0.04	0.835
Tannin	Light	0.15	0.705	6.89	**0.049**
	TSP Type	0.40	0.529	0.58	0.449
	NSR	0.08	0.778	0.57	0.483
	Light : TSP Type	0.11	0.740	0.37	0.543
	Light : NSR	0.54	0.461	5.81	**0.016**
	TSP Type : NSR	0.00	0.958	0.05	0.821
	Light : TSP Type : NSR	0.02	0.895	0.29	0.589
Leaf S	Light	5.96	**0.036**	0.30	0.611
	TSP Type	0.03	0.863	1.00	0.321
	NSR	0.01	0.915	2.66	0.110
	Light : TSP Type	1.49	0.223	0.03	0.857
	Light : NSR	1.34	0.247	0.58	0.447
	TSP Type : NSR	1.00	0.319	0.03	0.875
	Light : TSP Type : NSR	0.67	0.414	0.02	0.894
Leaf C	Light	2.71	0.129	22.26	**0.007**
	TSP Type	0.41	0.522	0.53	0.468
	NSR	0.09	0.768	0.63	0.442
	Light : TSP Type	1.15	0.285	1.38	0.241
	Light : NSR	1.49	0.223	2.33	0.128
	TSP Type : NSR	0.50	0.483	0.00	0.992
	Light : TSP Type : NSR	0.38	0.538	4.35	**0.038**
Cellulose	Light	2.84	0.126	0.05	0.835
	TSP Type	0.81	0.371	0.72	0.398
	NSR	0.01	0.929	0.45	0.510
	Light : TSP Type	0.00	0.955	0.49	0.483
	Light : NSR	0.96	0.327	1.97	0.161
	TSP Type : NSR	0.47	0.496	0.04	0.838
	Light : TSP Type : NSR	0.92	0.337	0.28	0.596
Lignin	Light	1.20	0.298	6.97	**0.039**
	TSP Type	1.37	0.251	0.59	0.446
	NSR	1.24	0.300	0.64	0.441
	Light : TSP Type	2.12	0.146	1.83	0.177
	Light : NSR	0.22	0.641	2.90	0.089
	TSP Type : NSR	0.00	0.965	2.71	0.106
	Light : TSP Type : NSR	0.54	0.465	0.10	0.756

		Deciduous species		Evergreen species	
**Trait**	**Predictor**	***F*-value**	***p*-value**	***F*-value**	***p*-value**
CN ratio	Light	0.00	0.985	25.75	**0.003**
	TSP Type	0.24	0.627	0.07	0.789
	NSR	0.50	0.487	0.21	0.654
	Light : TSP Type	4.62	**0.032**	8.02	**0.005**
	Light : NSR	5.70	**0.018**	1.03	0.310
	TSP Type : NSR	0.34	0.562	1.00	0.322
	Light : TSP Type : NSR	2.14	0.144	0.19	0.666
LDMC	Light	16.39	**0.002**	3.17	0.142
	TSP Type	0.08	0.772	0.07	0.793
	NSR	0.20	0.654	1.93	0.173
	Light : TSP Type	0.42	0.520	0.62	0.432
	Light : NSR	2.79	0.096	0.01	0.921
	TSP Type : NSR	0.00	0.989	0.07	0.786
	Light : TSP Type : NSR	0.43	0.513	1.04	0.308

References

Brach AR, Song H (2006) eFloras: New directions for online floras exemplified by the Flora of China Project. Taxon 55(1): 188-192

eFloras (2008). Published on the Internet http://www.efloras.org

Eichenberg D, Ristok C, Kröber W, Bruelheide H (2014) Plant polyphenols – implications of different sampling, storage and sample processing in biodiversity-ecosystem functioning experiments. Chem Ecol 30:676-692. doi: 10.1080/02757540.2014.894987

Graham HD (1992) Stabilization of the Prussian Blue Color in the Determination of Polyphenols. J Agric Food Chem 40:801-805. doi: 10.1021/jf00017a018

Hagerman AE (1987) Radial diffusion method for determining tannin in plant extracts. J Chem Ecol 13:437-449. doi: 0098-0331/87/0300-0437505.00/0

Murphy J, Riley JP (1958) A Single-Solution Method for the Determination of Soluble Phosphate in Sea Water. J Mar Biolog Assoc UK 37:9-14. doi: 10.1017/s0025315400014776

Pérez-Harguindeguy N et al. (2016) Corrigendum to: New handbook for standardised measurement of plant functional traits worldwide. Aust J Bot 64. doi: 10.1071/bt12225_co

Price ML, Butler LG (1977) Rapid Visual Estimation and Spectrophotometric Determination of Tannin Content of Sorghum Grain. J Agric Food Chem 25:1266-1273. doi: 10.1021/jf60214a034

Schinkel H (1984) Determination of Calcium, Magnesium, Strontium, Potassium, Sodium, Lithium, Iron, Manganese, Chromium, Nickel, Copper, Cobalt, Zinc and Cadmium by Flame AAS. A Universal Method for the Analysis of Waters, Coals, Ashes, Ores, Rocks, Building Materials, Metals and Similar Samples Fresenius Z Anal Chem 317:10-26. doi: 10.1007/BF00468391

Van Soest PJ, Robertson JB, Lewis BA (1991) Methods of Dietary Fiber, Neutral Detergent Fiber and Non-Starch Polysaccharides in Relation to Animal Nutrition. J Dairy Sci 74:3583 - 3597. doi: 10.3168/jds.S0022-0302(91)78551-2
